# Crosstalk between CD180-overexpression macrophages and glioma cells worsens patient survival through malignant phenotype promotion and immunosuppressive regulation

**DOI:** 10.1186/s10020-024-01029-w

**Published:** 2024-12-20

**Authors:** Wen Wang, Junsheng Li, Qiheng He, Chenglong Liu, Zhiyao Zheng, Bojian Zhang, Siqi Mou, Wei Sun, Jizong Zhao

**Affiliations:** 1 Department of Neurosurgery, Beijing Tiantan Hospital, Capital Medical University, No. 119 Nan Si Huan Xi Road, Fengtai District, Beijing, 100070 China; 2 China National Clinical Research Center for Neurological Diseases, Beijing, China

**Keywords:** CD180, Glioma, Macrophage, Immunotherapy, Biomarker

## Abstract

**Background:**

Understanding the molecular mechanisms in immunosuppressive regulation is crucial for improving immunotherapeutic strategies. Macrophages, the major immune cells in tumor microenvironment (TME), play a dual role in tumor progression. CD180, primarily expressed in macrophages, remains unclear and requires further investigation.

**Methods:**

RNA-seq data were obtained to analyze CD180 expression in gliomas and assess its prognostic value. The comprehensive immune infiltration analysis was performed. Single-cell RNA-seq (scRNA-seq) data were used to examine CD180 expression distribution at the cellular level. CD180-overexpression macrophages were co-cultured with three glioma cell lines. The effects on glioma cell behavior were evaluated through qRT-PCR, Western blot, CCK-8 assay, EdU assay, Transwell assay, TUNEL assay, and flow cytometry. Differentially expressed genes (DEGs) and their potential biological functions were analyzed between different CD180 expression groups. Consensus clustering was used to identify CD180-related glioma subtypes.

**Results:**

CD180 was significantly upregulated in glioma samples and associated with poor prognosis. High CD180 expression was correlated with increased immune cell infiltration, particularly macrophages, and elevated levels of immune checkpoints. Analysis of scRNA-seq data revealed the predominant expression of CD180 in macrophages within the glioma TME. In vitro experiments demonstrated that CD180-overexpression macrophages promoted glioma cell proliferation, migration, invasion, and epithelial-mesenchymal transition (EMT), while decreasing apoptosis. Mutations in TP53 and PTEN were significantly more prevalent in the high CD180 expression group. We identified nine chemotherapeutic agents that were more effective in glioma patients with high CD180 expression. Additionally, two CD180-related glioma subtypes with distinct prognosis were identified.

**Conclusions:**

This study confirmed the prognostic role of CD180 in glioma and its involvement in immunosuppressive regulation and malignant phenotype promotion. Therefore, CD180 was considered as a promising target for immunotherapeutic strategies in glioma treatment.

**Supplementary Information:**

The online version contains supplementary material available at 10.1186/s10020-024-01029-w.

## Background

Gliomas are the most common primary brain tumors and present significant clinical challenges (Nicholson and Fine 2021). Despite advancements in treatment, overall survival (OS) remains unfavorable, particularly for high-grade gliomas (Kirby and Finnerty 2021). The molecular characteristics of gliomas can enhance our understanding of glioma biology and help monitor treatment responses (Barthel et al. 2022). Key molecular biomarkers, such as IDH mutation, 1p/19q codeletion, and MGMT promoter methylation, have been extensively studied (Hofer and Lassman 2010). The World Health Organization (WHO) has integrated molecular diagnostics into glioma classification. Molecular evaluation is important for prognosis and personalized treatment (Berger et al. 2022). However, accurately predicting prognosis of gliomas remains challenging due to the cellular and molecular heterogeneity. Further research is needed to delineate molecular subtypes.

TME includes various cellular and non-cellular components that interact dynamically with tumor cells. It is a complex ecosystem that influences tumor progression (Visser and Joyce 2023). The critical role of TME, especially tumor immune microenvironment (TIME), in modulating tumor behavior and therapeutic response has been recognized (Bejarano et al. 2021). Immune cells are the major component of TIME, and they could play a dual role in tumor progression. The balance between pro-inflammatory and immunosuppressive states determines the efficacy of anti-tumor responses and therapeutic effects (Pansy et al. 2021). Immunotherapy has revolutionized tumor treatment. It demonstrates remarkable clinical efficacy in certain patient subsets. However, it is highly dependent on the composition and functional status of TIME (Walsh and Quail 2023). Strategies aimed at reprogramming TIME could enhance anti-tumor immunity, modulate immune checkpoint pathways, and overcome immunosuppressive mechanisms. Therefore, understanding the molecular and cellular mechanisms governing TIME interactions and identifying prognostic biomarkers are essential for improving the efficacy of immunotherapy.

CD180 is also known as RP105. It is a member of Toll-like receptor (TLR) family and plays a crucial role in the innate immune response (Edwards et al. 2023). CD180 has been studied in inflammatory and autoimmune diseases. Its role in gliomas is less understood. Emerging evidence suggests that CD180 acts as a specific inhibitor of TLR4 signaling in macrophages and inhibits the activation of macrophages and dendritic cells (DCs) (Fan et al. 2021). It promotes the accumulation and immunosuppressive activity of myeloid-derived suppressor cells (MDSCs) (Dong et al. 2019). Furthermore, CD180 is significantly upregulated in tumor-associated macrophages (TAMs) (Gottfried et al. 2003). These findings suggest that CD180 may influence tumor progression and patient prognosis through immunosuppressive effects.

**Fig. 1:**
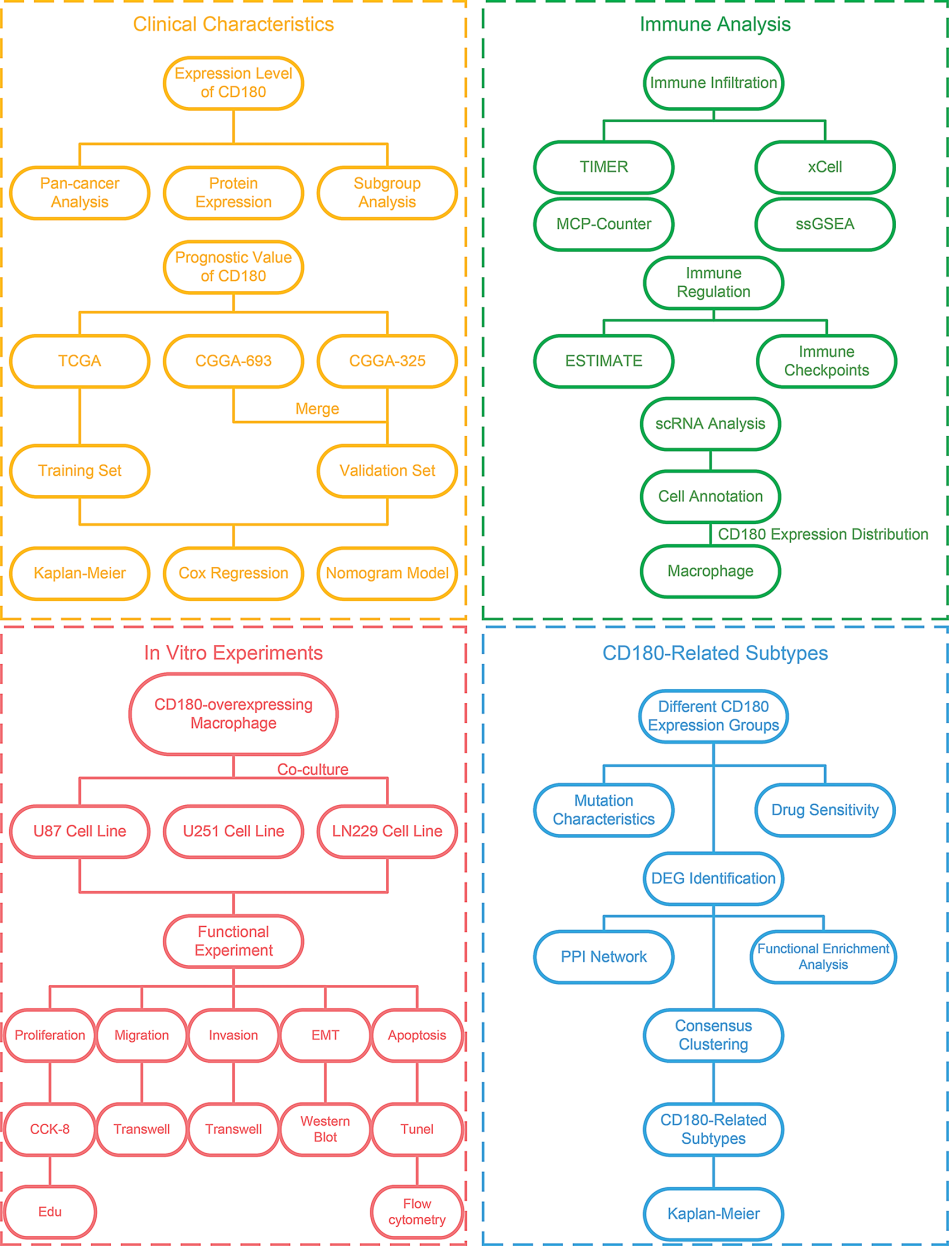
Flowchart of the study design

In this study, the expression patterns and prognostic value of CD180 were analyzed using 2296 samples from five independent datasets, and the comprehensive immune infiltration analysis of CD180 was conducted. The scRNA-seq revealed that CD180 was primarily expressed in macrophages of glioma samples. We co-cultured three glioma cell lines with CD180-overexpression macrophages to assess the effects on glioma cell proliferation, migration, invasion, EMT, and apoptosis. We identified potential chemotherapeutic agents that might be effective in gliomas with high CD180 expression. Moreover, we identified DEGs between different CD180 expression groups and classified glioma samples into two CD180-related subtypes. The flowchart of our study has been shown (Fig. 1).

## Methods

### Data collection and processing

RNA-seq data of pan-cancer in TPM format, uniformly processed by Toil pipeline, were obtained from UCSC XENA platform (https://xenabrowser.net/datapages/) (Goldman et al. 2020). We collected the RNA-seq and corresponding clinical information from TCGA database (https://portal.gdc.cancer.gov/) as the training set (McKenna et al. 2010). Two RNA-seq datasets (CGGA-693, CGGA-325) sourced from CGGA database (http://www.cgga.org.cn/) (Zhao et al. 2021) were adjusted for batch effects using the ComBat function of sva package, and merged as the validation set (Leek et al. 2012). Further validation utilized the CGGA-301 dataset from CGGA database and the GSE16011 dataset from GEO database (https://www.ncbi.nlm.nih.gov/geo/) (Barrett et al. 2013). Protein expression of CD180 in glioma was obtained from UALCAN database (http://ualcan.path.uab.edu/index.html/) (Chandrashekar et al. 2017).

### Survival analysis and nomogram establishment

Kaplan-Meier curves were conducted to examine survival distribution discrepancies between different CD180 expression groups in glioma samples. Patients with complete clinical data were included for Cox regression analysis to confirm the significance of CD180 expression in prognostic prediction. These clinical characteristics were then integrated to develop a nomogram model, with its predictive efficacy evaluated using time-dependent ROC curves and calibration plots. Prognostic value of CD180 in gliomas was validated using three external datasets.

### Immune characteristics analysis

Association between CD180 expression and immune cell infiltration levels in glioma samples was initially investigated using TIMER database (https://cistrome.shinyapps.io/timer/) (Li et al. 2017). Moreover, xCell and MCP-counter algorithms were performed to analyze immune infiltration differences between different CD180 expression groups (Aran et al. 2017; Becht et al. 2016). Additionally, ssGSEA algorithm was used to identify correlations between CD180 expression and infiltration levels of 24 immune cells (Hänzelmann et al. 2013; Bindea et al. 2013). Associations between CD180 expression and Immune Score, Stromal Score, ESTIMATE Score, and Tumor Purity were also analyzed (Yoshihara et al. 2013).

### Analysis of scRNA-seq data

We obtained the scRNA-seq data of glioma samples from GSE182109 dataset (Abdelfattah et al. 2022). The Seurat package was utilized to analyze the raw scRNA-seq data. Quality control involved filtering out genes present in fewer than 3 cells or cells expressing fewer than 300 genes, more than 9000 genes, more than 15% mitochondrial genes, or more than 1% hemoglobin genes. After data normalization, identification of variable genes, and integration of data from multiple samples, we scaled the data and performed principal component analysis (PCA). Cells were grouped using UMAP clustering with a resolution of 0.8 and annotated with known marker genes.

### Cell recovery and culture

To recover target cells, we pre-warmed ddH2O to 37 °C and retrieve the cryopreservation tube containing the cells from liquid nitrogen tank. The tube was gently shaken to thaw the cells within 2–3 min, then transferred to a centrifuge tube and centrifuged at 1000 rpm for 5 min at room temperature. The supernatant was carefully aspirated, and the cell pellet was resuspended. Cells were cultured in a T25 flask (430639, Corning, USA) in an incubator set to 37 °C with 5% CO_2_.

### Induction and treatment for macrophages

THP-1 cells were cultured to a density of 80–90% and resuspended at a concentration of 5 × 10^5^ cells/mL with RPMI-1640 complete medium. PMA was added at a concentration of 100 ng/mL, and cells were cultured for 72 h. After 24 h, the supernatant was discarded, and cells were washed three times with PBS. RPMI-1640 complete medium was added, and cells were cultured until reaching about 80% confluence. M0 macrophages were collected and seeded into culture flasks, then transfected with CD180 lentivirus and control virus. The medium was changed after 24 h, replacing it with RPMI-1640 complete medium.

### Co-culture model

We used transwell chambers to construct a co-culture model. Glioma cell lines were seeded in the lower layer of the chamber. Treated macrophages were seeded in the upper layer of the chamber. After co-culture for an appropriate period of time, the cells in the lower layer were collected for subsequent experiments.

### Quantitative real-time polymerase chain reaction (qRT-PCR)

Total RNA was extracted from cells using TRIzol (#15596-018; Ambion). The PrimeScrip RT Reagent Kit (RR047; Takara, Japan) and TB Green Premix Ex Taq (RR820; Takara, Japan) were used for cDNA reverse transcription, and the qRT-PCR was performed using StepOnePlus System (Life, Singapore). Specific primers for genes have been described (Table S1). ΔΔCt method was performed to determine the relative expression of genes at transcriptome level, and GAPDH was selected as the reference gene.

### Western blot assay

Protein samples were collected using radioimmunoprecipitation assay (RIPA) lysis buffer (P0013B, Beyotime) mixed with Protease Inhibitor Cocktail (CW2200, CWBIO) and Phosphatase Inhibitor Cocktail (CW2383, CWBIO). The samples were incubated on ice for 30 min and quantified using the Pierce™ BCA Protein Assay Kit (23227, Thermo Scientific). Equivalent amounts of protein were separated by electrophoresis and transferred onto 0.45 μm–0.25 μm polyvinylidene fluoride (PVDF) membranes (IPVH00010 and ISEQ00010, Immobilon). Membranes were blocked with Bovine Serum Albumin (A6020, Biotopped) for 1 h at room temperature and then incubated with primary antibodies overnight at 4 °C. Bands were visualized using ECL detection reagent (MA0186-2, Meilunbio). The antibodies used have been listed (Table S2).

### Proliferation assay

For CCK-8 assay, cells were plated into a 96-well plate at 2000 cells/well. After attachment, the CCK-8 reagent (CA1210, Solarbio) was added and recorded as day zero. The absorbance values were measured using a microplate reader (3020–81480, Thermo) at 450 nm at 24, 48, and 96 h. For EdU assay, appropriate numbers of co-cultured tumor cells were placed in 6-well plates. Add an appropriate amount of EdU (C0078, Beyotime) according to the instructions, and complete labeling after 2 h. Cells were fixed and further stained according to the manufacturer guidance.

### Transwell assay

For transwell migration assay, 200 ul serum-free medium and cells were added to the transwell chamber, which was then placed in the incubator for over 30 min. Following this, 500 ul serum was added to the lower chamber for culture. For transwell invasion assay, 100–200 µl diluted Matrigel (1 mg/ml) (M8370, Solarbio) was added to the bottom of the upper chamber and incubated at 37 °C for 4–5 h to dry into a gel. Add 500 ul serum to the lower chamber, slowly add cells along the inner wall of the upper chamber, and collect samples after 24 h. The results were analyzed by Wilcoxon rank-sum test.

### TUNEL assay

Cells were cultured to a suitable density, washed with PBS, and fixed with 4% paraformaldehyde (P1110, Solarbio) for 30 min. After washing, cells were treated with PBS containing 0.3% Triton X-100 at room temperature for 5 min. 50 µl TUNEL detection solution was added, and samples were incubated at 37ºC in the dark for 60 min. After washing, cells were observed under a fluorescence microscope (DM3000, Leica).

### Flow cytometric assay

Cultured cells were digested with an appropriate amount of trypsin and resuspended in a centrifuge tube. Adjust the cells to a suitable density, and add Annexin V-FITC binding solution. After mixing, Annexin V-FITC and propidium iodide staining solution were added successively. Following incubation at room temperature, samples were tested on the flow cytometric device.

### Mutation landscape and drug sensitivity

The top 10 mutated genes in the different CD180 expression groups were identified, respectively. Subsequently, the frequencies of the top 10 mutated genes in glioma were compared between two groups. Based on the Genomics of Drug Sensitivity in Cancer (GDSC) database, we used the oncopredict package to evaluate the drug sensitivity of each sample and assess the response to chemotherapeutic drugs. Several common chemotherapeutic drugs were selected according to their sensitivity (Maeser et al. 2021).

### Differential expression analysis and function enrichment analysis

We identified the differentially expressed mRNAs between two groups using limma package. The DEGs were defined with the screening criteria of adj. P value less than 0.05 and logFC value over 2. The protein-protein interaction (PPI) network of these genes was established using STRING database (https://cn.string-db.org/) with a minimum interaction score threshold set at 0.4 (Szklarczyk et al. 2023). Subsequently, the network was imported and visualized using Cytoscape software. Functional enrichment analyses, including GO and KEGG analysis, were conducted using ClusterProfiler package to explore their potential biological functions (Yu et al. 2012).

### Consensus clustering

Consensus clustering was performed based on the DEGs to classify samples into distinct clusters using ConsensuClusterPlus package (Wilkerson and Hayes 2010). This process involved 500 bootstrapping iterations utilizing the km method and employing Canberra as the metric distance. Each bootstrap included 80% of the samples. The optimal number of molecular subtypes was determined by calculating the consistency matrix and consistency cumulative distribution function (CDF). PCA was then performed to visualize and differentiate the clusters.

### Statistical analysis

In this study, the R project was used for statistical analyses. Wilcoxon rank-sum tests were used for two-group comparisons, while Kruskal-Wallis tests were applied for comparisons involving more than two groups. Spearman correlation tests assessed correlations between two variables. A two-sided P-value of less than 0.05 was considered statistically significant for all analyses.

## Results

### Expression characteristics of CD180

**Fig. 2:**
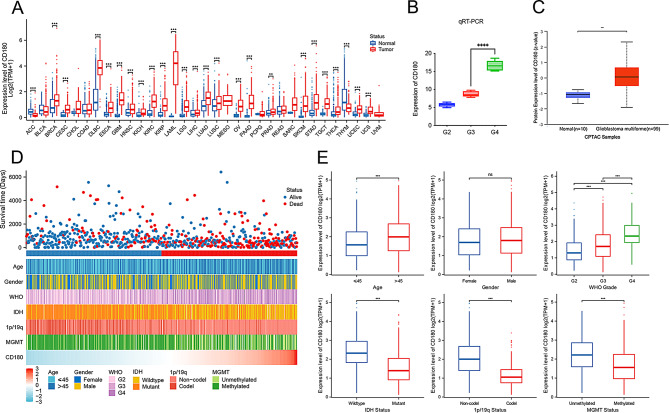
Comprehensive analysis of CD180 expression pattern. *, *P* < 0.05; **, *P* < 0.01; ***, *P* < 0.001; ns, not significant. (**A**) Pan-cancer analysis of CD180 expression in normal tissues and tumor samples. (**B**) CD180 expression levels in different glioma grades using qRT-PCR. (**C**) Protein expression of CD180 in normal tissues and glioma samples in CPTAC dataset from UALCAN database. (**D**) Landscape of CD180-related clinicopathological features of gliomas. (**E**) CD180 expression levels in different clinical subgroups

The expression level of CD180 was first investigated in pan-cancer, and it showed that CD180 exhibited significantly higher expression levels in tumor samples compared to normal tissues for most cancer types (Fig. 2A). Subsequently, we validated the expression of CD180 using qRT-PCR in an independent glioma group (Fig. 2B). We also observed a significant upregulation of CD180 protein expression in CPTAC dataset from UALCAN database (Fig. 2C). The distinct patterns of clinicopathologic features associated with CD180 were identified, including asymmetric distributions of WHO grades, IDH mutation, 1p/19q codeletion, and MGMT methylation (Fig. 2D). Moreover, we compared the expression level of CD180 between different clinical subgroups. It showed that CD180 significantly increased with the grades of glioma. Samples in subgroups of IDH wildtype, 1p/19q non-codeletion, and MGMT promoter unmethylation showed significantly higher expression of CD180 (Fig. 2E). These findings suggested CD180 could enhance the malignancy of gliomas.

### Prognostic role of CD180 in glioma

**Fig. 3:**
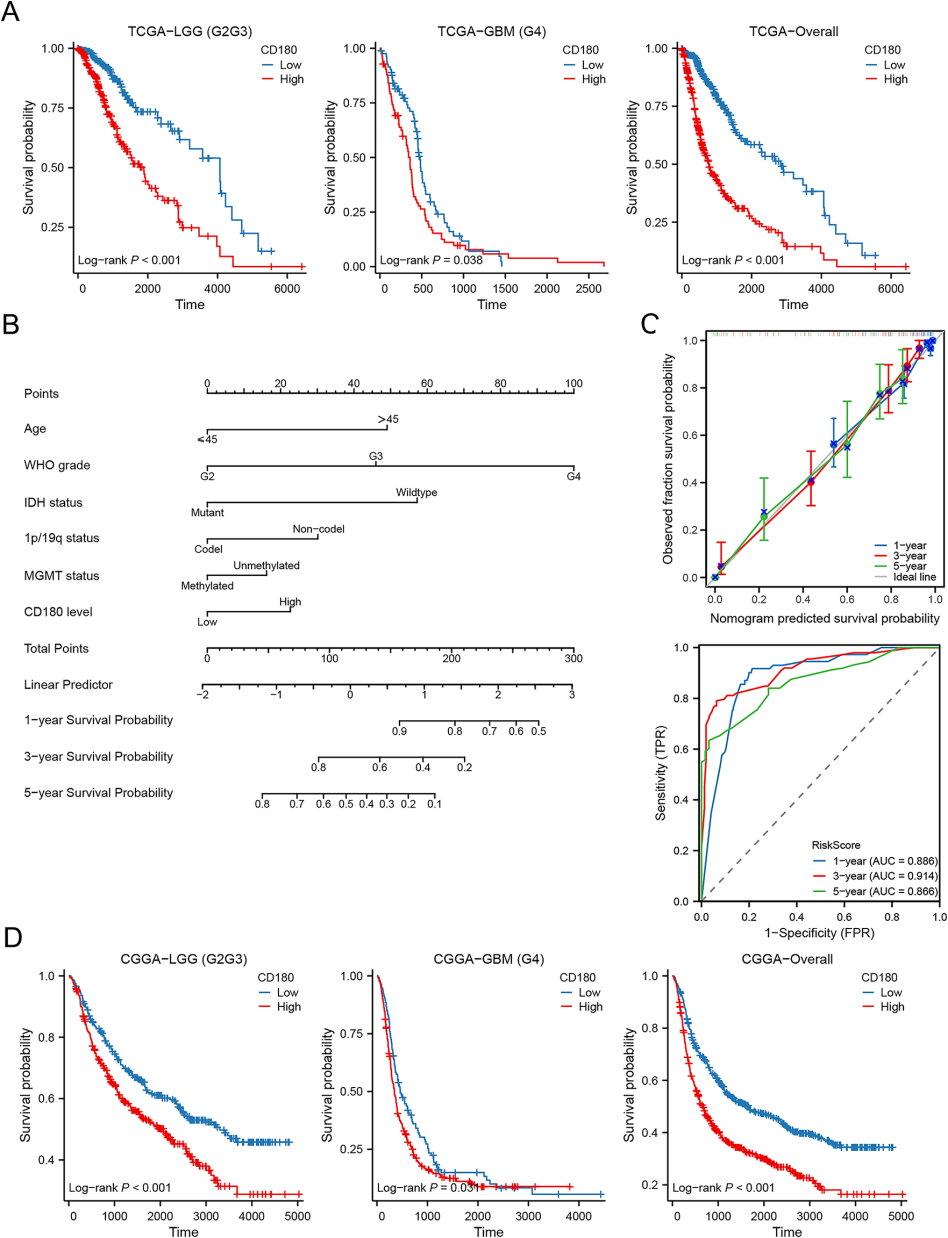
Prognostic role of CD180 in glioma. (**A**) Kaplan-Meier analysis for different CD180 expression groups in TCGA database. (**B**) Nomogram model for survival prediction. (**C**) Time-dependent ROC curves and calibration plots. (**D**) Validation of survival distribution between different CD180 expression groups in CGGA database

Survival analysis indicated that high CD180 expression was significantly associated with a worse prognosis (*P* < 0.05 for all, Fig. 3A). Cox regression identified CD180 as an independent prognostic factor for glioma patients (HR = 1.454, 95%CI = 1.046–2.020, *P* = 0.026, Table 1). We further developed a prognostic nomogram for better clinical application (C-index = 0.849, 95%CI = 0.837–0.862, Fig. 3B). Time-dependent ROC curves and calibration plots supported the favorable predictive accuracy of the nomogram (Fig. 3C). Additionally, we confirmed the prognostic value of CD180 in the validation set. It showed that glioma patients with high CD180 expression correlated with a significantly shorter OS (*P* < 0.05 for all, Fig. 3D). CD180 was validated as an independent prognostic factor in the validation set (Table 2). To further reinforce our findings, the results were consistent that CD180 was a poor prognosis biomarker for glioma in CGGA-301 and GSE16011 datasets (*P* < 0.05 for both, Fig. S1).

**Table 1:** Cox regression of TCGA dataset

Characteristics	Univariate analysis		Multivariate analysis	
	Hazard ratio (95% CI)	*P* value	Hazard ratio (95% CI)	*P* value
Age				
≤ 45	Reference		Reference	
> 45	4.308 (3.165–5.862)	< 0.001*	2.249 (1.523–3.323)	< 0.001*
Gender				
Female	Reference			
Male	1.103 (0.838–1.450)	0.484		
WHO grade				
G2	Reference		Reference	
G3	3.404 (2.294–5.051)	< 0.001*	2.139 (1.413–3.237)	< 0.001*
G4	22.438 (14.564–34.568)	< 0.001*	5.222 (3.097–8.807)	< 0.001*
IDH status				
Wildtype	Reference		Reference	
Mutant	0.101 (0.075–0.136)	< 0.001*	0.388 (0.242–0.623)	< 0.001*
1p/19q status				
Non-codel	Reference		Reference	
Codel	0.243 (0.155–0.382)	< 0.001*	0.607 (0.356–1.036)	0.067
MGMT status				
Unmethylated	Reference		Reference	
Methylated	0.294 (0.223–0.388)	< 0.001*	0.766 (0.551–1.065)	0.112
CD180 level				
Low	Reference		Reference	
High	3.106 (2.318–4.163)	< 0.001*	1.454 (1.046–2.020)	0.026*

*P < 0.05, significant difference

**Table 2:** Cox regression of CGGA dataset

Characteristics	Univariate analysis		Multivariate analysis	
	Hazard ratio (95% CI)	*P* value	Hazard ratio (95% CI)	*P* value
Age				
≤ 45	Reference		Reference	
> 45	1.799 (1.501–2.156)	< 0.001*	1.221 (1.003–1.487)	0.047*
Gender				
Female	Reference			
Male	1.076 (0.897–1.291)	0.429		
WHO grade				
G2	Reference		Reference	
G3	2.801 (2.094–3.745)	< 0.001*	2.669 (1.990–3.579)	< 0.001*
G4	7.432 (5.609–9.848)	< 0.001*	4.966 (3.645–6.765)	< 0.001*
IDH status				
Wildtype	Reference		Reference	
Mutant	0.337 (0.280–0.406)	< 0.001*	0.842 (0.668–1.061)	0.144
1p/19q status				
Non-codel	Reference		Reference	
Codel	0.236 (0.175–0.318)	< 0.001*	0.375 (0.273–0.517)	< 0.001*
MGMT status				
Unmethylated	Reference		Reference	
Methylated	0.803 (0.671–0.960)	0.016*	0.899 (0.747–1.082)	0.260
CD180 level				
Low	Reference		Reference	
High	1.689 (1.409–2.026)	< 0.001*	1.232 (1.018–1.490)	0.032*

*P < 0.05, significant difference

### Immune characteristics of CD180

**Fig. 4:**
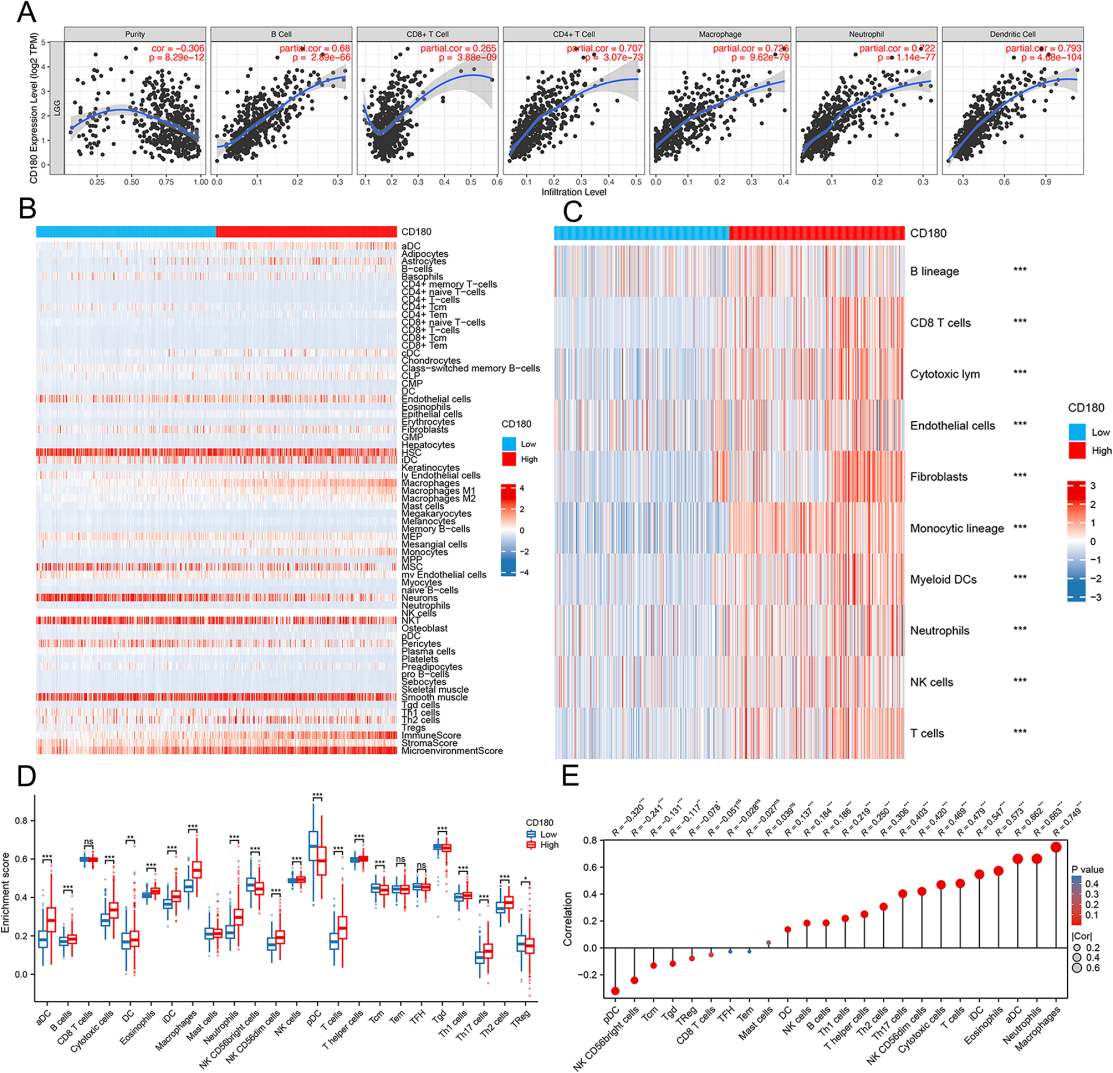
Association between immune characteristics and CD180 expression in glioma. (**A**) Correlation between CD180 expression and immune infiltration in TIMER database. (**B**) Immune infiltration levels in different CD180 expression groups by xCell. (**C**) Immune infiltration levels in different CD180 expression groups by MCP-counter. (**D**) Immune infiltration levels in different CD180 expression groups by ssGSEA. (**E**) Correlation between CD180 expression and infiltration levels of 24 immune cells

**Fig. 5:**
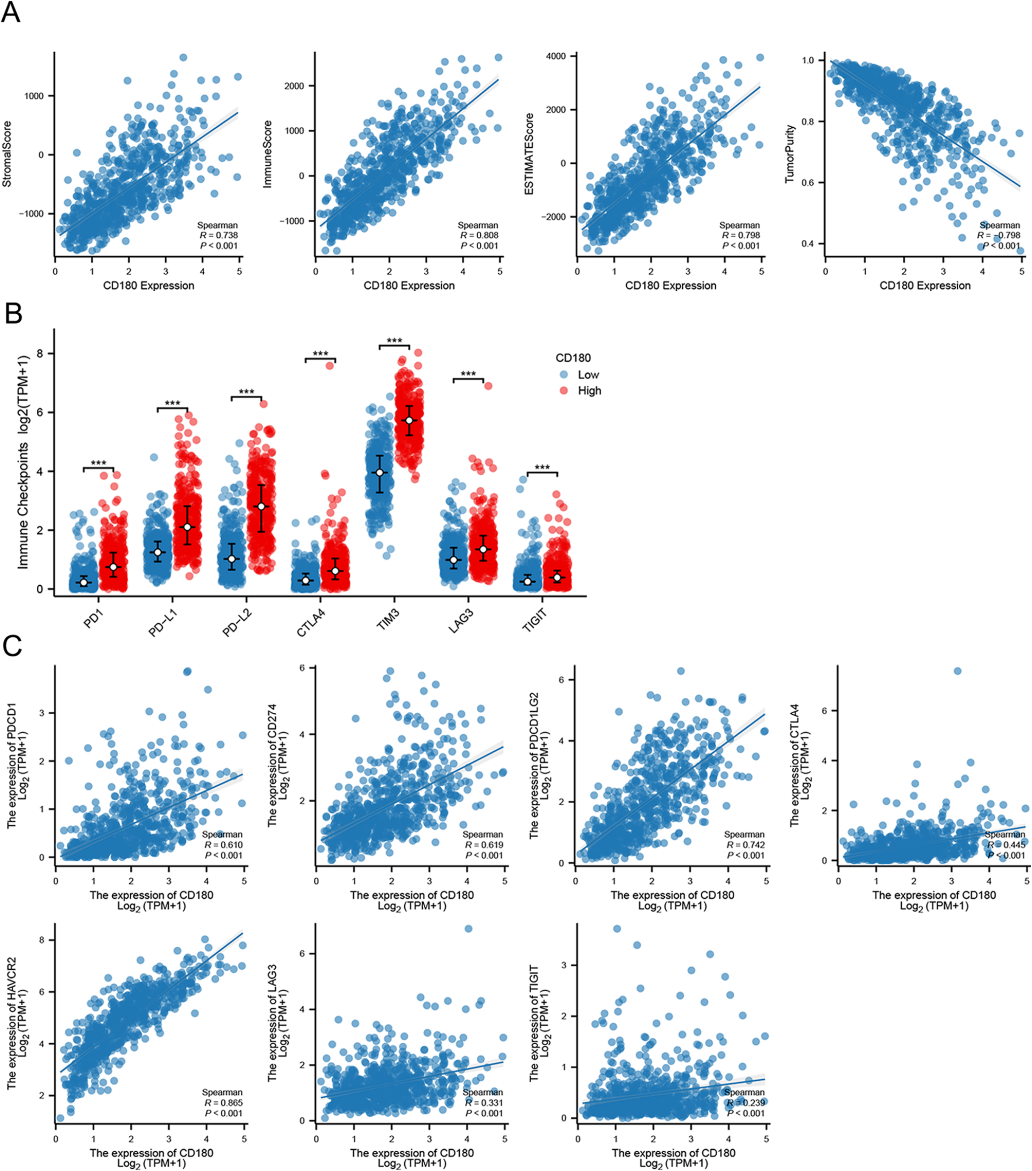
Association between immune regulation and CD180 expression in glioma. (**A**) Correlation between CD180 expression and stromal score, immune score, ESTIMATE score, and tumor purity. (**B**) Expression levels of immune checkpoints in different CD180 expression groups. (**C**) Correlation between CD180 expression and immune checkpoints

**Fig. 6:**
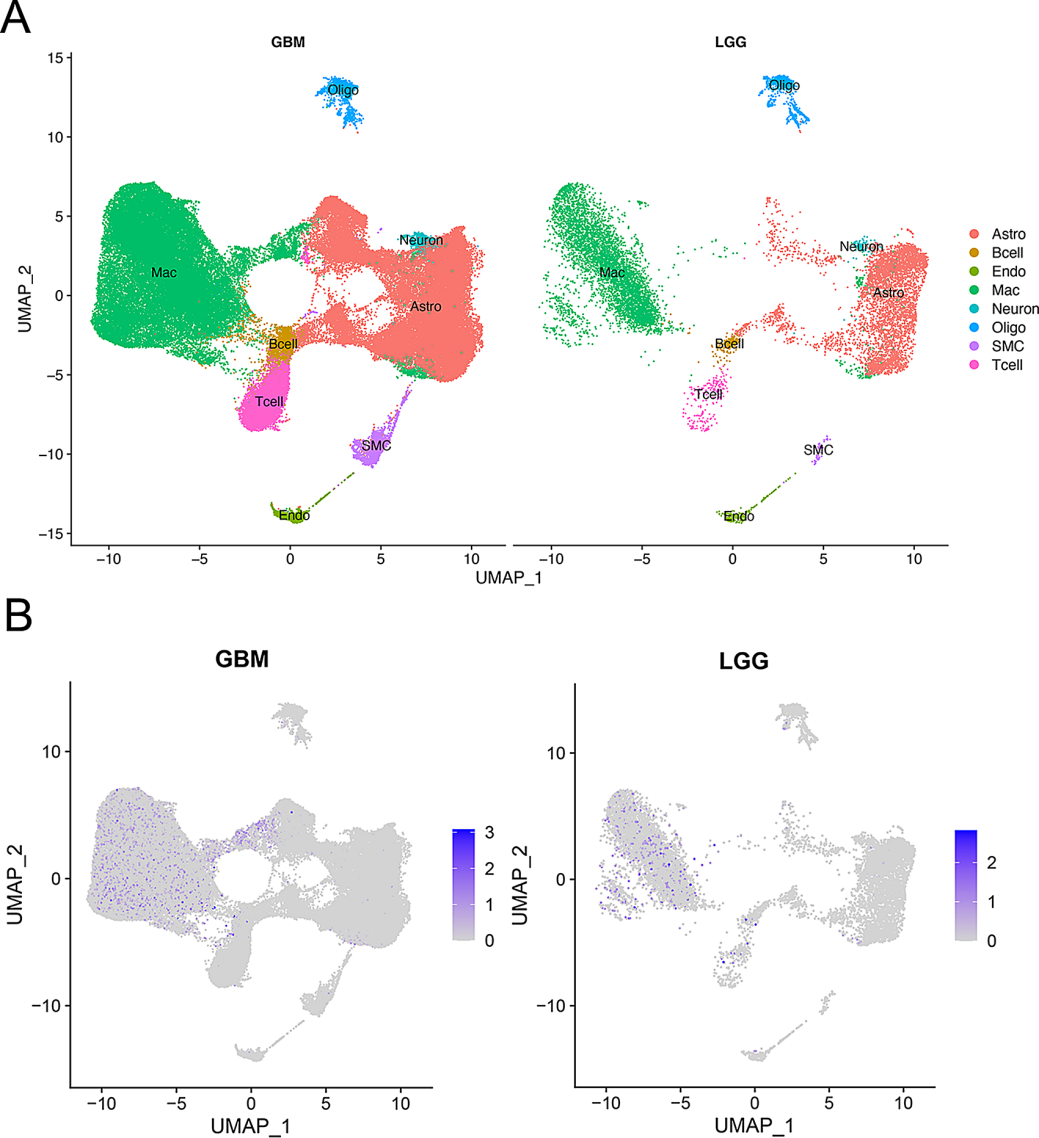
scRNA-seq analysis of CD180 expression distribution in glioma microenvironment. (**A**) Annotation of cell subsets in lower-grade glioma (LGG) and glioblastoma (GBM). (**B**) Distribution of CD180 expression within various cell subsets. CD180 was predominantly expressed in macrophages

Based on TIMER database, we found that CD180 was positively correlated with the infiltration level of immune cells in glioma (Fig. 4A). The results of xCell and MCP-counter analyses verified that immune infiltration levels were elevated in the high CD180 expression group (Fig. 4B-C). It suggested that a variety of immune cells were elevated in the high CD180 expression group using ssGSEA analysis (Fig. 4D). Especially, the expression level of CD180 was associated with macrophage infiltration level in glioma (Fig. 4E). ESTIMATE analysis showed that CD180 was significantly positively correlated with stromal score, immune score, and ESTIMATE score, but negatively correlated with tumor purity, suggesting the potential role of CD180 in TME (Fig. 5A). We further analyzed the correlation between the expression level of CD180 and major immune checkpoints. The expression levels of immune checkpoints were significantly upregulated in high CD180 expression group (Fig. 5B), and it presented a consistently positive correlation (Fig. 5C). scRNA-seq analysis was performed to examine the expression of CD180 at cell level. It showed that CD180 was predominantly expressed in macrophages, suggesting an immunosuppressive effect in the TME of gliomas (Fig. 6).

### Role of CD180-overexpression macrophages in glioma

**Fig. 7:**
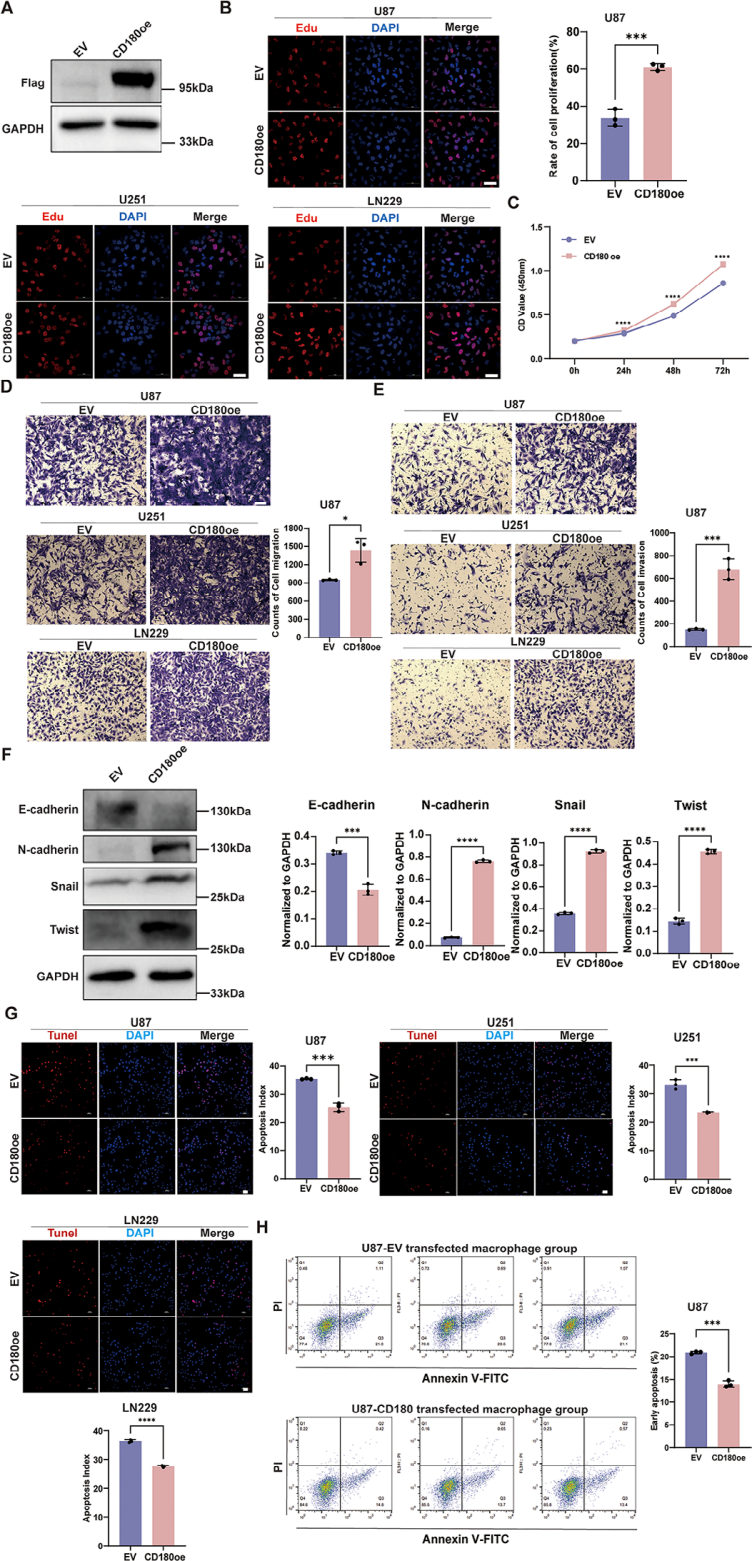
CD180-overexpression macrophages promoted the proliferation, migration, invasion and EMT while decreasing apoptosis of glioma in vitro. *, *P* < 0.05; **, *P* < 0.01; ***, *P* < 0.001; ****, *P* < 0.0001. (**A**) Western blot assay showing successful expression of Flag protein in CD180 overexpression group. (**B**) Representative image of Edu assay. Scale bar, 50 μm. Each experiment was replicated for three times. (**C**) Line graph showing CCK-8 assays at 24, 48, and 72 h after co-culture in U87 cell line. The cell proliferation ability was significantly enhanced in both EdU and CCK-8 assays after co-cultured with CD180-transfected macrophages. (**D**-**E**) Representative image of transwell migration and invasion assays in U87, U251, and LN229 cell line. Scale bar, 100 μm. Each experiment was replicated for three times. The cell migration and invasion ability were significantly enhanced after co-cultured with CD180-transfected macrophages. (**F**) Western blot images (left) and histograms (right) of E-cadherin, N-cadherin, Snail, and Twist in U87 cell line subjected to different treatments. Each experiment was replicated for three times. The expression level of E-cadherin was significantly downregulated, while N-cadherin, Snail, and Twist were significantly upregulated. (**G**) Representative image of TUNEL assay in U87, U251, and LN229 cell line. Scale bar, 50 μm. Each experiment was replicated for three times. (**H**) Annexin V-PI flow cytometry analysis in U87 cell line before and after co-culture. The apoptosis ability is significantly reduced in both TUNEL and V-PI flow cytometry assays after co-cultured with CD180-transfected macrophages

To further explore the role of CD180-overexpression macrophages in the progression of glioma, macrophages induced from THP-1 were transfected with CD180 overexpressing lentivirus. The results showed that CD180 was successfully overexpressed at both transcriptomic and protein levels (Fig. 7A, Fig. S2). Then the CD180-transfected macrophages were co-cultured with glioma cell lines including U87, U251 and LN229. After U87, U251 and LN229 were respectively co-cultured with CD180-transfected macrophages, compared with the empty vehicle group, the cell proliferation ability was significantly enhanced in both EdU and CCK-8 assays (*P* < 0.05, Fig. 7B-C, Fig. S3-4). Transwell migration and invasion assays were performed to evaluate cellular behavior, and the results showed significantly enhanced ability of migration and invasion of glioma cell lines after co-cultured with CD180-transfected macrophages (*P* < 0.05, Fig. 7D-E). In addition to mesenchymal behaviors such as migration, we also examined the markers related to EMT at the protein level using western blotting. The results showed that after co-culture, the expression level of E-cadherin was significantly downregulated, while N-cadherin, Snail, and Twist were significantly upregulated (*P* < 0.05, Fig. 7F, Fig. S5). Moreover, we performed TUNEL and Annexin V-PI flow cytometry assays to detect the apoptosis ability in glioma cell lines. In glioma cell lines co-cultured with CD180-transfected macrophages, the apoptosis ability is significantly reduced (Fig. 7G). Notably, through flow cytometry analysis, we found that although both early apoptotic and late apoptotic cells were reduced, the decrease in early apoptotic cells was dominant (Fig. 7H, Fig. S6).

### Mutation characteristics and drug sensitivity of CD180

**Fig. 8:**
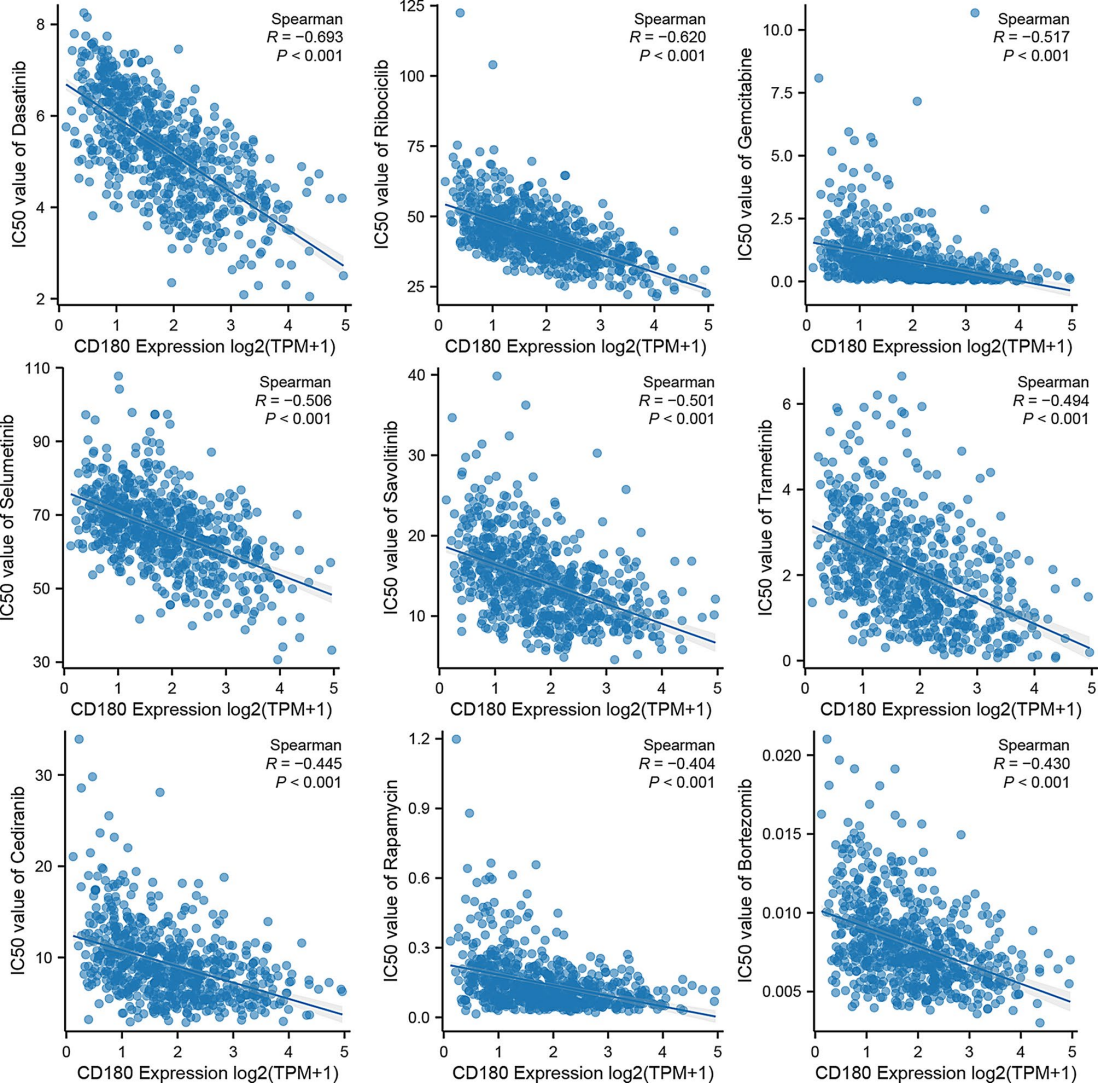
Correlation between CD180 expression and different chemotherapeutic agents. Chemotherapeutic agents (Dasatinib, Ribociclib, Gemcitabine, Selumetinib, Savolitinib, Trametinib, Cediranib, Rapamycin, Bortezomib) could potentially exhibit enhanced efficacy in gliomas with high CD180 expression

We identified the top 10 mutant genes in low CD180 expression and high CD180 expression groups, respectively. And we further compared the prevalence of the top 10 mutant genes in gliomas (Fig. S7). It showed that the frequencies of TP53, EGFR, and PTEN mutations were significantly higher in the high CD180 expression group, whereas the frequency of IDH1 mutation was significantly lower (*P* < 0.05 for all). Moreover, we identified the top 9 common chemotherapeutic agents that glioma patients with high CD180 expression might be sensitive. We evaluated the correlation between CD180 expression and drug sensitivity of these agents (Fig. 8). It suggested the therapeutic potential of these drugs in the treatment of glioma with high CD180 expression.

### Identification of DEGs and functional enrichment analysis

**Fig. 9:**
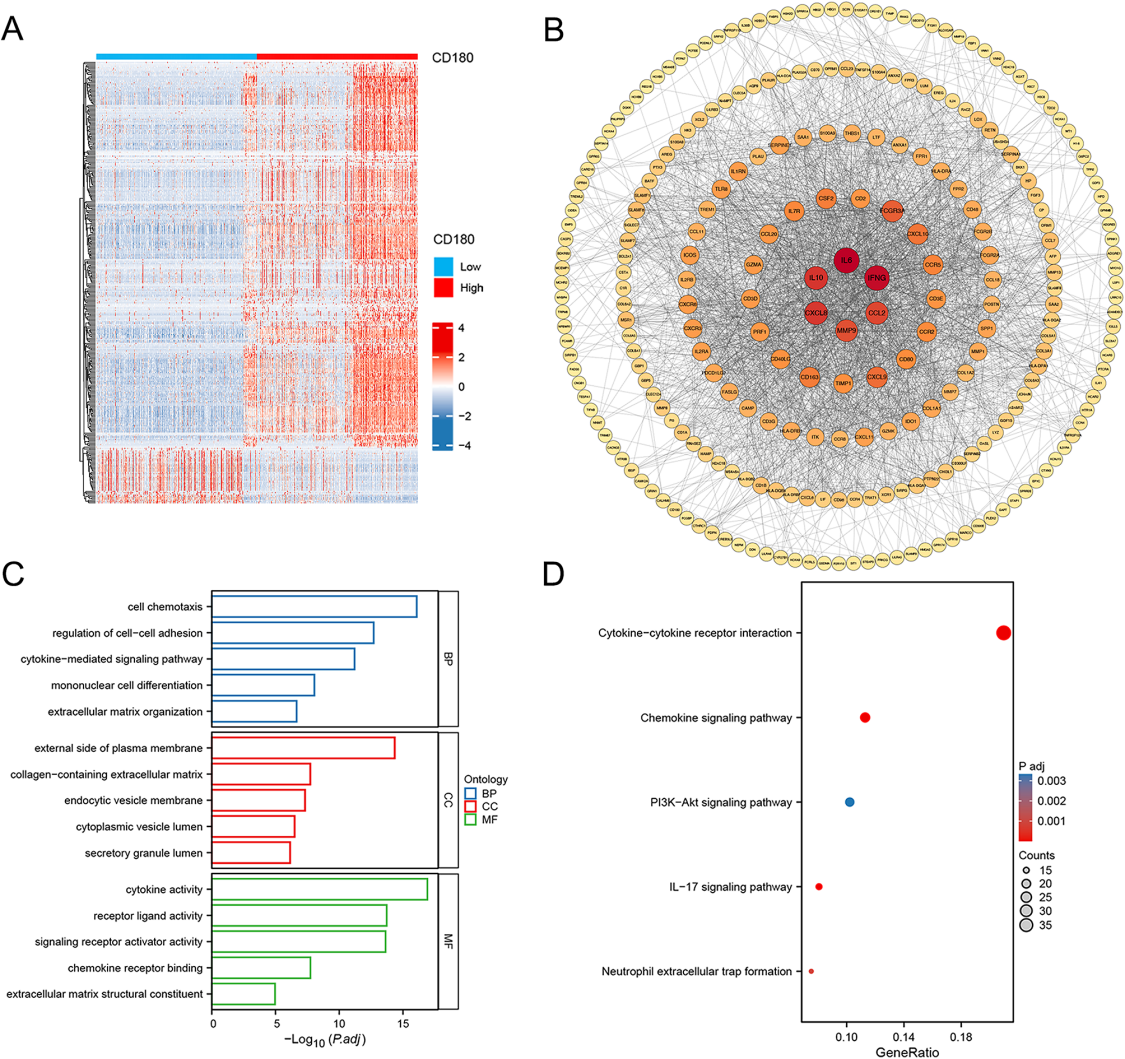
Integrated analysis of DEGs and potential biological functions. (**A**) Heatmap of DEG expression. (**B**) Construction of PPI network. (**C**) GO analysis. (**D**) KEGG analysis

We identified 349 DEGs between different CD180 expression groups and visualized the expression patterns of these genes using a heatmap (Fig. 9A). To further understand the genetic interactions, we constructed a PPI network and ranked the genes based on the node degree, highlighting the genetic interactions (Fig. 9B). Functional enrichment analyses were conducted to explore the potential biological functions of these genes. GO analysis indicated that these genes were primarily associated with biological processes, such as cell chemotaxis, regulation of cell-cell adhesion, cytokine-mediated signaling pathways, mononuclear cell differentiation, and extracellular matrix organization (Fig. 9C). Similarly, KEGG pathway analysis revealed that these genes are predominantly involved in pathways, such as cytokine-cytokine receptor interaction, chemokine signaling pathway, PI3K-Akt signaling pathway, IL-17 signaling pathway, and neutrophil extracellular trap formation (Fig. 9D).

### Classification of CD180-related subtypes in glioma

**Fig. 10:**
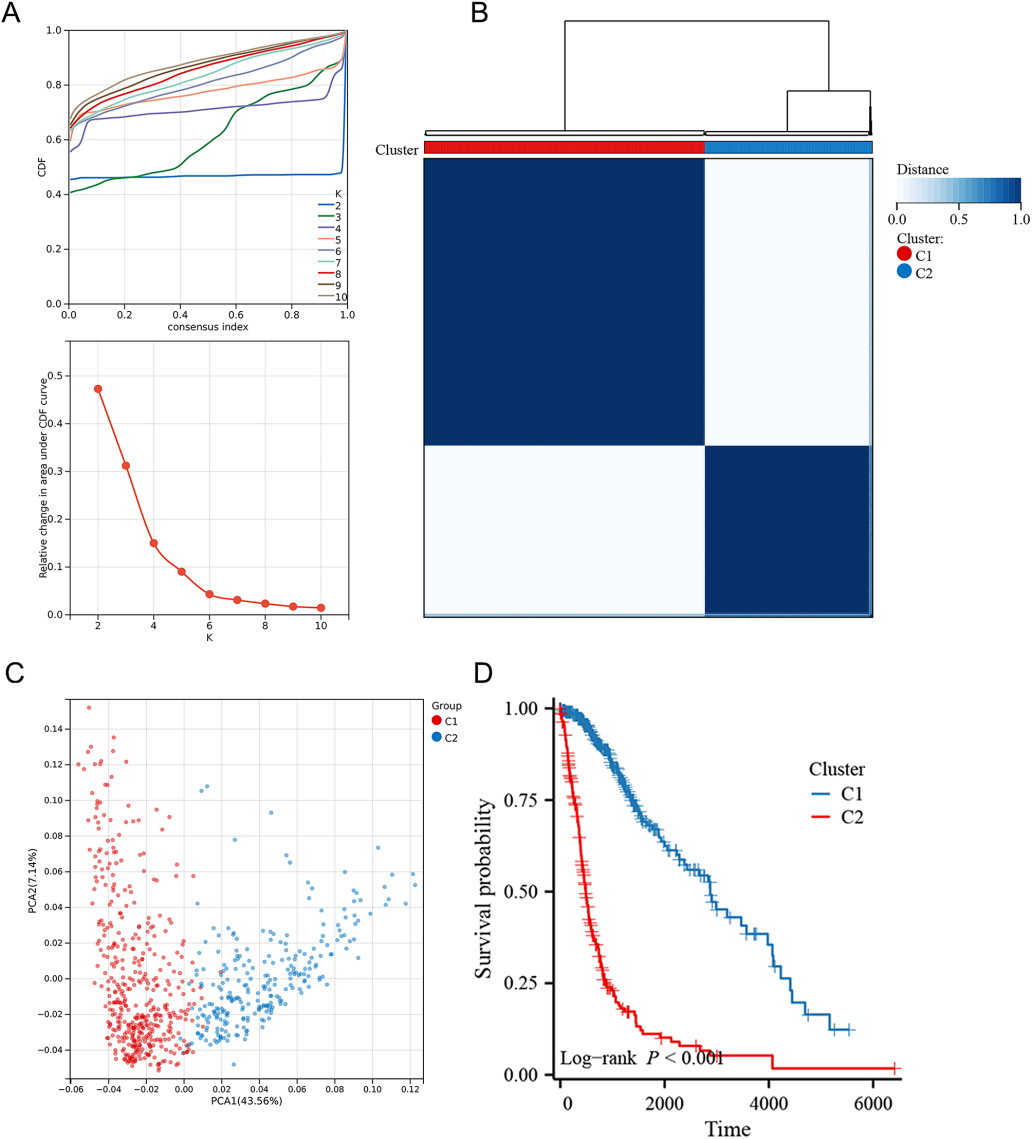
Identification of CD180-related subtypes in glioma. (**A**) CDF curves for consensus scores and CDF delta area curves. (**B**) Identification of two CD180-related subtypes by consensus clustering analyses. (**C**) Distinction of two CD180-related subtypes by PCA. (**D**) Survival distribution between two CD180-related subtypes

Given the intrinsic heterogeneity within tumor, we performed consensus clustering analysis to categorize glioma samples into distinct subtypes based on the DEGs. According to CDF and CDF Delta area curve, we identified the optimal number of clusters as 2 (Fig. 10A). We found that samples were classified into two robust clusters, designated as cluster 1 and cluster 2 (Fig. 10B). PCA algorithm further validated this classification, revealing two markedly distinct components. This clear separation indicated that the samples were effectively divided into two distinct groups (Fig. 10C). Additionally, we examined the survival distribution between these two subtypes. The results demonstrated a significantly worse prognosis for cluster 2 compared to cluster 1, highlighting the prognostic significance of CD180 in gliomas (Fig. 10D).

## Discussion

Glioma is characterized by the aggressive growth and heterogeneity. It presents significant therapeutic challenges. Clinical outcomes for glioma patients remain suboptimal, due to the functional deficits caused by surgical operations and resistance to chemoradiotherapy (Alifieris and Trafalis 2015). Exploring new treatment strategy is a worldwide challenge. Immunotherapy has emerged as promising strategy, it could provide durable responses and improve survival rates in glioma patients (Xu et al. 2020). However, the efficacy of immunotherapy critically depends on the dynamic landscape of TME.

Within the TME, macrophages are the most abundant immune cells and exhibit significant plasticity. Macrophages could differentiate into various phenotypes in response to different stimuli (Christofides et al. 2022). The roles are multifaceted, contributing to tumor suppression and promotion. Macrophages produce pro-inflammatory cytokines, promote Th1 responses, and activate cytotoxic T cells, inhibiting tumor growth (Chen et al. 2023). Conversely, macrophages express immune checkpoints, produce anti-inflammatory cytokines, growth factors and matrix metalloproteinases (MMPs), facilitating tissue repair, angiogenesis, and immunosuppression, which promote tumor growth and invasion (Duan and Luo 2021). Thus, reprogramming macrophages to adopt anti-tumorigenic functions while inhibiting their pro-tumorigenic activities, presents a potential strategy to enhance immunotherapy efficacy (Xia et al. 2020).

The glioma cellular microenvironment is dominated by TAMs. It demonstrates TAMs secret growth factors supporting glioma stem cell self-renewal (Buonfiglioli and Hambardzumyan 2021). TAMs exert important role in glioma proliferation, migration and invasion (Bettinger et al. 2002). TAM cell state transitions correlate with glioma progression (Yeo et al. 2022). TAMs present a compelling treatment target in glioma (Majety et al. 2018). It reported that inhibition of CCL2/CCR2 axis could reduce tumor infiltration and stimulate T cell responses (Chang et al. 2016). Knockdown CD73 presents survival benefits in glioma (Azambuja et al. 2020). CAR-Macrophages could recognize tumor-specific surface antigens (Chen et al. 2022) and it may infiltrate glioblastoma more effectively (Pan et al. 2022). Moreover, CD47 antibody combined with alkylating chemotherapy has proved effective in glioblastoma (Roemeling et al. 2020).

CD180 is known for its role in cell activation and modulation of immunoglobulin production. It is highly expressed in antigen presenting cells and regulates the action of classical TLRs with various other receptors (Edwards et al. 2023). Previous studies have revealed the immunosuppressive role of CD180 in macrophages. Compared to macrophages cultured without tumor cell contact, CD180 expression was significantly upregulated in TAMs (Gottfried et al. 2003). CD180 expression was markedly increased in monocytes in response to microbial stimulation (Zarember and Godowski 2002). It inhibited macrophage activation by modulating TLR4 signaling and exhibited negative regulation of TNF-α and IL-12 secretion (Liu et al. 2013). Moreover, CD180 promoted the accumulation of MDSCs and enhances their immunosuppressive activity on M1 macrophage polarization (Dong et al. 2019). CD180 deficiency could result in an increased local inflammatory response (Wezel et al. 2016). These findings suggested CD180 in macrophages could alleviate inflammation. CD180 has also been identified as a prognostic biomarker in hematological malignancies and esophageal squamous cell carcinoma (Kramer et al. 2022; Wang et al. 2022). However, no studies have yet investigated the role of CD180 in gliomas, indicating a critical area for future research.

In this study, we found that CD180 was significantly upregulated in gliomas at both RNA and protein levels. Subsequently, we analyzed the relationship between CD180 expression and clinicopathologic features. It showed that the elevated CD180 expression in gliomas was associated with more aggressive clinical subgroups, suggesting its role in tumor malignancy. Therefore, we further explored the prognostic value of CD180 in glioma. High CD180 expression was correlated with a worse prognosis in glioma patients. Cox regression analysis confirmed the independence of CD180. The validation of these findings across multiple datasets underscored the robustness of CD180 as a prognostic biomarker in gliomas. The immune characteristics of CD180 revealed its significant association with immune cell infiltration in gliomas. Specifically, high CD180 expression was correlated with increased infiltration of various immune cells, particularly macrophages. The results of scRNA-seq analysis confirmed that CD180 was concentrated in macrophages within gliomas. This was consistent with previous studies. The positive correlation between CD180 expression and immune scores and stromal score, alongside the negative correlation with tumor purity, also suggested the potential role of CD180 in shaping TME. The upregulation of immune checkpoints in high CD180 expression groups implied that CD180 might contribute to an immunosuppressive TME, which could facilitate immune evasion by tumor cells. These findings indicated the involvement of CD180 in immunosuppressive modulation and tumor progression.

We performed in vitro experiments to elucidate the role of CD180-overexpression macrophages within the glioma microenvironment. CD180-overexpression macrophages enhanced glioma cell proliferation, migration, and invasion while reducing apoptotic capacities. These results supported the hypothesis that CD180 contributed to an immunosuppressive and pro-tumorigenic microenvironment. The significant enhancement in cellular behaviors promoted tumor aggressiveness, so CD180 could be a potential therapeutic target for gliomas. One of the central findings was the alteration in EMT marker expression, where E-cadherin was downregulated, N-cadherin, Snail, and Twist were upregulated after co-culture. The modulation of these markers often associated with a more aggressive malignant behavior of tumor cells (Lu and Kang 2019; Noronha et al. 2021). The elucidation of this molecular shift provided a plausible pathway, through which CD180-overexpression macrophages might facilitate glioma progression. Apoptotic assays revealed reduced apoptosis in glioma cells after co-culture. CD180 might exert protective effects on glioma cells at an early stage of the apoptotic pathway, due to the early apoptotic events were predominantly reduced. This anti-apoptotic influence contributed to the survival and accumulation of malignant cells within the TME, further exacerbating disease progression. As CD180 is a transmembrane protein, its effect could be mediated either through direct receptor-ligand interactions or by secreted factors. It reported that CD180-mediated signaling lead to downregulation of the immunosuppressive IL-10 secretion level (Shcherbina et al. 2021). Moreover, the EMT marker expression was altered after co-culture with CD180-transfected macrophages. Specifically, we propose that CD180 overexpression in macrophages may affect the secretion of immunomodulatory cytokines (e.g., IL-10, TGF-β) that suppress anti-tumor immunity and promote glioma cell proliferation.

Mutation analysis revealed distinct mutation profiles between different CD180 expression groups. It implies a potential association between CD180 expression and specific genetic alterations that propel glioma progression. Furthermore, drug sensitivity analysis identified several chemotherapeutic agents that could potentially exhibit enhanced efficacy in gliomas with high CD180 expression, thereby emphasizing promising strategies for chemotherapy. To explore the impact of CD180 in tumor heterogeneity, we classified the glioma samples into two distinct CD180-related glioma subtypes. Two clusters revealed significant difference in survival outcomes. This classification provided a new perspective on glioma heterogeneity. These findings highlighted the potential stratifying role of CD180 and suggested the implementation of personalized treatment strategies in clinical practice. Despite these insights, this study has several limitations that warrant acknowledgment. Firstly, it relied on retrospective analyses, emphasizing the necessity for validation of the prognostic role of CD180 in large-scale, multi-center prospective cohorts. Additionally, we acknowledge that in vitro models, while useful, do not fully replicate the complexity of the glioma tumor microenvironment. As our study relies on co-culture systems, we have provided a mechanistic insight into the interaction between CD180-overexpression macrophages and glioma cells. However, it is important to recognize that factors like blood-brain barrier dynamics, the heterogeneity of the TME, and interactions with other immune cells cannot be fully recapitulated in vitro. In vivo models, including orthotopic glioma mouse models, will be crucial to further validate the translational relevance of our findings. In these models, the complexity of immune infiltration and glioma-stroma interactions could be better captured. Such studies could more accurately simulate therapeutic outcomes and prognostic biomarkers in a clinical setting.

## Conclusions

This study revealed the association between CD180 expression and unfavorable clinical outcomes in gliomas, highlighting CD180 as a potential prognostic biomarker. Additionally, our investigation also delved into the role of CD180 in immunosuppressive regulation and malignant phenotype promotion in glioma. These findings enhance our understanding of glioma biology and hold considerable promise for the development of innovative immunotherapeutic strategies in glioma treatment.

## Electronic supplementary material

Below is the link to the electronic supplementary material.

Supplementary Material 1

## Data Availability

Data supporting the conclusion in this study were available from the corresponding authors with reasonable requests, including raw and processed data.

## Abbreviations

OS: Overall survival
IDH: Isocitrate dehydrogenase
MGMT: O6-methylguanine DNA methyltranferase
WHO: World Health Organization, TME, Tumor microenvironment
TIME: Tumor immune microenvironment
TLR: Toll-like receptor
DC: Dendritic cell
MDSC: Myeloid-derived suppressor cell
TAM: Tumor-associated macrophage
scRNA-seq: Single-cell RNA sequencing
EMT: Epithelial-mesenchymal transition
DEG: Differentially expressed gene, TCGA, The Cancer Genome Atlas
CGGA: Chinese Glioma Genome Atlas
GEO: Gene Expression Omnibus
qRT-PCR: Quantitative real-time polymerase chain reaction
GDSC: Genomics of Drug Sensitivity in Cancer
PPI: Protein-protein interaction
CDF: Cumulative distribution function
GO: Gene Ontology
KEGG: Kyoto Encyclopedia of Genes and Genomes
MMP: Matrix metalloproteinase

## References

[CR26] Abdelfattah N, Kumar P, Wang C, Leu JS, Flynn WF, Gao R, et al. Single-cell analysis of human glioma and immune cells identifies S100A4 as an immunotherapy target. Nat Commun. 2022;13(1):767. 10.1038/s41467-022-28372-y. 10.1038/s41467-022-28372-y PMC8828877 35140215

[CR31] Alifieris C, Trafalis DT. Glioblastoma multiforme: Pathogenesis and treatment. Pharmacol Ther. 2015;152:63–82. 10.1016/j.pharmthera.2015.05.005. 25944528 10.1016/j.pharmthera.2015.05.005

[CR21] Aran D, Hu Z, Butte AJ. xCell: digitally portraying the tissue cellular heterogeneity landscape. Genome Biol. 2017;18(1):220. 10.1186/s13059-017-1349-1. 29141660 10.1186/s13059-017-1349-1 PMC5688663

[CR42] Azambuja JH, Schuh RS, Michels LR, Iser IC, Beckenkamp LR, Roliano GG, et al. Blockade of CD73 delays glioblastoma growth by modulating the immune environment. Cancer Immunol Immunotherapy: CII. 2020;69(9):1801–12. 10.1007/s00262-020-02569-w. 32350590 10.1007/s00262-020-02569-w PMC11027675

[CR18] Barrett T, Wilhite SE, Ledoux P, Evangelista C, Kim IF, Tomashevsky M, et al. NCBI GEO: archive for functional genomics data sets–update. Nucleic Acids Res. 2013;991–5. 10.1093/nar/gks1193. 41 Database issue:D. 10.1093/nar/gks1193 PMC3531084 23193258

[CR3] Barthel L, Hadamitzky M, Dammann P, Schedlowski M, Sure U, Thakur BK, et al. Glioma: molecular signature and crossroads with tumor microenvironment. Cancer Metastasis Rev. 2022;41(1):53–75. 10.1007/s10555-021-09997-9. 34687436 10.1007/s10555-021-09997-9 PMC8924130

[CR22] Becht E, Giraldo NA, Lacroix L, Buttard B, Elarouci N, Petitprez F, et al. Estimating the population abundance of tissue-infiltrating immune and stromal cell populations using gene expression. Genome Biol. 2016;17(1):218. 10.1186/s13059-016-1070-5. 10.1186/s13059-016-1113-y PMC5134277 27908289

[CR7] Bejarano L, Jordāo MJC, Joyce JA. Therapeutic targeting of the Tumor Microenvironment. Cancer Discov. 2021;11(4):933–59. 10.1158/2159-8290.cd-20-1808. 33811125 10.1158/2159-8290.CD-20-1808

[CR5] Berger TR, Wen PY, Lang-Orsini M, Chukwueke UN. World Health Organization 2021 Classification of Central Nervous System Tumors and implications for Therapy for adult-type gliomas: a review. JAMA Oncol. 2022;8(10):1493–501. 10.1001/jamaoncol.2022.2844. 36006639 10.1001/jamaoncol.2022.2844

[CR38] Bettinger I, Thanos S, Paulus W. Microglia promote glioma migration. Acta Neuropathol. 2002;103(4):351–5. 10.1007/s00401-001-0472-x. 11904754 10.1007/s00401-001-0472-x

[CR24] Bindea G, Mlecnik B, Tosolini M, Kirilovsky A, Waldner M, Obenauf AC, et al. Spatiotemporal dynamics of intratumoral immune cells reveal the immune landscape in human cancer. Immunity. 2013;39(4):782–95. 10.1016/j.immuni.2013.10.003. 24138885 10.1016/j.immuni.2013.10.003

[CR37] Buonfiglioli A, Hambardzumyan D. Macrophages and microglia: the cerberus of glioblastoma. Acta Neuropathol Commun. 2021;9(1):54. 10.1186/s40478-021-01156-z. 33766119 10.1186/s40478-021-01156-z PMC7992800

[CR19] Chandrashekar DS, Bashel B, Balasubramanya SAH, Creighton CJ, Ponce-Rodriguez I, Chakravarthi B, et al. UALCAN: a portal for facilitating Tumor Subgroup Gene expression and survival analyses. Neoplasia (New York NY). 2017;19(8):649–58. 10.1016/j.neo.2017.05.002. 10.1016/j.neo.2017.05.002 PMC5516091 28732212

[CR41] Chang AL, Miska J, Wainwright DA, Dey M, Rivetta CV, Yu D, et al. CCL2 produced by the Glioma Microenvironment is essential for the Recruitment of Regulatory T Cells and myeloid-derived suppressor cells. Cancer Res. 2016;76(19):5671–82. 10.1158/0008-5472.can-16-0144. 27530322 10.1158/0008-5472.CAN-16-0144 PMC5050119

[CR43] Chen C, Jing W, Chen Y, Wang G, Abdalla M, Gao L, et al. Intracavity generation of glioma stem cell-specific CAR macrophages primes locoregional immunity for postoperative glioblastoma therapy. Sci Transl Med. 2022;14(656):eabn1128. 10.1126/scitranslmed.abn1128. 35921473 10.1126/scitranslmed.abn1128

[CR34] Chen S, Saeed A, Liu Q, Jiang Q, Xu H, Xiao GG, et al. Macrophages in immunoregulation and therapeutics. Signal Transduct Target Therapy. 2023;8(1):207. 10.1038/s41392-023-01452-1. 10.1038/s41392-023-01452-1 PMC10200802 37211559

[CR33] Christofides A, Strauss L, Yeo A, Cao C, Charest A, Boussiotis VA. The complex role of tumor-infiltrating macrophages. Nat Immunol. 2022;23(8):1148–56. 10.1038/s41590-022-01267-2. 35879449 10.1038/s41590-022-01267-2 PMC10754321

[CR6] de Visser KE, Joyce JA. The evolving tumor microenvironment: from cancer initiation to metastatic outgrowth. Cancer Cell. 2023;41(3):374–403. 10.1016/j.ccell.2023.02.016. 36917948 10.1016/j.ccell.2023.02.016

[CR12] Dong G, Yao X, Yan F, Zhang H, Zhu Y, Yang Y, et al. Ligation of CD180 contributes to endotoxic shock by regulating the accumulation and immunosuppressive activity of myeloid-derived suppressor cells through STAT3. Biochim et Biophys acta Mol Basis Disease. 2019;1865(3):535–46. 10.1016/j.bbadis.2018.12.013. 10.1016/j.bbadis.2018.12.013 30557700

[CR35] Duan Z, Luo Y. Targeting macrophages in cancer immunotherapy. Signal Transduct Target Therapy. 2021;6(1):127. 10.1038/s41392-021-00506-6. 10.1038/s41392-021-00506-6 PMC7994399 33767177

[CR10] Edwards K, Lydyard PM, Kulikova N, Tsertsvadze T, Volpi EV, Chiorazzi N, et al. The role of CD180 in hematological malignancies and inflammatory disorders. Mol Med (Cambridge Mass). 2023;29(1):97. 10.1186/s10020-023-00682-x. 10.1186/s10020-023-00682-x PMC10353253 37460961

[CR11] Fan Z, Pathak JL, Ge L. The potential role of RP105 in regulation of inflammation and Osteoclastogenesis during Inflammatory diseases. Front cell Dev Biology. 2021;9:713254. 10.3389/fcell.2021.713254. 10.3389/fcell.2021.713254 PMC8369417 34414191

[CR14] Goldman MJ, Craft B, Hastie M, Repečka K, McDade F, Kamath A, et al. Visualizing and interpreting cancer genomics data via the Xena platform. Nat Biotechnol. 2020;38(6):675–8. 10.1038/s41587-020-0546-8. 32444850 10.1038/s41587-020-0546-8 PMC7386072

[CR13] Gottfried E, Faust S, Fritsche J, Kunz-Schughart LA, Andreesen R, Miyake K, et al. Identification of genes expressed in tumor-associated macrophages. Immunobiology. 2003;207(5):351–9. 10.1078/0171-2985-00246. 14575150 10.1078/0171-2985-00246

[CR23] Hänzelmann S, Castelo R, Guinney J. BMC Bioinformatics. 2013;14:7. 10.1186/1471-2105-14-7. GSVA: gene set variation analysis for microarray and RNA-seq data. 10.1186/1471-2105-14-7 PMC3618321 23323831

[CR4] Hofer S, Lassman AB. Molecular markers in gliomas: impact for the clinician. Target Oncol. 2010;5(3):201–10. 10.1007/s11523-010-0157-2. 20809335 10.1007/s11523-010-0157-2

[CR2] Kirby AJ, Finnerty GT. New strategies for managing adult gliomas. J Neurol. 2021;268(10):3666–74. 10.1007/s00415-020-09884-3. 32542524 10.1007/s00415-020-09884-3 PMC8463358

[CR49] Kramer MH, Zhang Q, Sprung R, Day RB, Erdmann-Gilmore P, Li Y, et al. Proteomic and phosphoproteomic landscapes of acute myeloid leukemia. Blood. 2022;140(13):1533–48. 10.1182/blood.2022016033. 35895896 10.1182/blood.2022016033 PMC9523374

[CR17] Leek JT, Johnson WE, Parker HS, Jaffe AE, Storey JD. The sva package for removing batch effects and other unwanted variation in high-throughput experiments. Bioinf (Oxford England). 2012;28(6):882–3. 10.1093/bioinformatics/bts034. 10.1093/bioinformatics/bts034 PMC3307112 22257669

[CR20] Li T, Fan J, Wang B, Traugh N, Chen Q, Liu JS, et al. TIMER: a web server for Comprehensive Analysis of Tumor-infiltrating Immune cells. Cancer Res. 2017;77(21):e108–10. 10.1158/0008-5472.can-17-0307. 29092952 10.1158/0008-5472.CAN-17-0307 PMC6042652

[CR47] Liu B, Zhang N, Liu Z, Fu Y, Feng S, Wang S, et al. RP105 involved in activation of mouse macrophages via TLR2 and TLR4 signaling. Mol Cell Biochem. 2013;378(1-2):183–93. 10.1007/s11010-013-1609-7. 23483427 10.1007/s11010-013-1609-7

[CR51] Lu W, Kang Y. Epithelial-mesenchymal plasticity in Cancer Progression and Metastasis. Dev Cell. 2019;49(3):361–74. 10.1016/j.devcel.2019.04.010. 31063755 10.1016/j.devcel.2019.04.010 PMC6506183

[CR27] Maeser D, Gruener RF, Huang RS. oncoPredict: an R package for predicting in vivo or cancer patient drug response and biomarkers from cell line screening data. Brief Bioinform. 2021;22(6). 10.1093/bib/bbab260. 10.1093/bib/bbab260 PMC8574972 34260682

[CR40] Majety M, Runza V, Lehmann C, Hoves S, Ries CH. A drug development perspective on targeting tumor-associated myeloid cells. FEBS J. 2018;285(4):763–76. 10.1111/febs.14277. 28941174 10.1111/febs.14277

[CR15] McKenna A, Hanna M, Banks E, Sivachenko A, Cibulskis K, Kernytsky A, et al. Genome Res. 2010;20(9):1297–303. 10.1101/gr.107524.110. The Genome Analysis Toolkit: a MapReduce framework for analyzing next-generation DNA sequencing data. 10.1101/gr.107524.110 PMC2928508 20644199

[CR1] Nicholson JG, Fine HA. Diffuse glioma heterogeneity and its therapeutic implications. Cancer Discov. 2021;11(3):575–90. 10.1158/2159-8290.cd-20-1474. 33558264 10.1158/2159-8290.CD-20-1474

[CR52] Noronha C, Ribeiro AS, Taipa R, Castro DS, Reis J, Faria C, et al. Cadherin expression and EMT: a Focus on Gliomas. Biomedicines. 2021;9:10. 10.3390/biomedicines9101328. 10.3390/biomedicines9101328 PMC8533397 34680444

[CR44] Pan K, Farrukh H, Chittepu V, Xu H, Pan CX, Zhu Z. CAR race to cancer immunotherapy: from CAR T, CAR NK to CAR macrophage therapy. J Experimental Clin cancer Research: CR. 2022;41(1):119. 10.1186/s13046-022-02327-z. 10.1186/s13046-022-02327-z PMC8969382 35361234

[CR8] Pansy K, Uhl B, Krstic J, Szmyra M, Fechter K, Santiso A, et al. Immune Regulatory processes of the Tumor Microenvironment under Malignant conditions. Int J Mol Sci. 2021;22:24. 10.3390/ijms222413311. 10.3390/ijms222413311 PMC8706102 34948104

[CR53] Shcherbina V, Gordiienko I, Shlapatska L, Gluzman D, Sidorenko S. CD150 and CD180 are negative regulators of IL-10 expression and secretion in chronic lymphocytic leukemia B cells. Neoplasma. 2021;68(4):760–9. 10.4149/neo_2021_210104N8. 33904315 10.4149/neo_2021_210104N8

[CR28] Szklarczyk D, Kirsch R, Koutrouli M, Nastou K, Mehryary F, Hachilif R, et al. The STRING database in 2023: protein-protein association networks and functional enrichment analyses for any sequenced genome of interest. Nucleic Acids Res. 2023;51(D1):D638–46. 10.1093/nar/gkac1000. 36370105 10.1093/nar/gkac1000 PMC9825434

[CR45] von Roemeling CA, Wang Y, Qie Y, Yuan H, Zhao H, Liu X, et al. Therapeutic modulation of phagocytosis in glioblastoma can activate both innate and adaptive antitumour immunity. Nat Commun. 2020;11(1):1508. 10.1038/s41467-020-15129-8. 10.1038/s41467-020-15129-8 PMC7083893 32198351

[CR9] Walsh LA, Quail DF. Decoding the tumor microenvironment with spatial technologies. Nat Immunol. 2023;24(12):1982–93. 10.1038/s41590-023-01678-9. 38012408 10.1038/s41590-023-01678-9

[CR50] Wang D, Dai J, Suo C, Wang S, Zhang Y, Chen X. Molecular subtyping of esophageal squamous cell carcinoma by large-scale transcriptional profiling: characterization, therapeutic targets, and prognostic value. Front Genet. 2022;13:1033214. 10.3389/fgene.2022.1033214. 36425064 10.3389/fgene.2022.1033214 PMC9678939

[CR48] Wezel A, de Vries MR, Maassen JM, Kip P, Peters EA, Karper JC, et al. Deficiency of the TLR4 analogue RP105 aggravates vein graft disease by inducing a pro-inflammatory response. Sci Rep. 2016;6:24248. 10.1038/srep24248. 27053419 10.1038/srep24248 PMC4823661

[CR30] Wilkerson MD, Hayes DN. ConsensusClusterPlus: a class discovery tool with confidence assessments and item tracking. Bioinf (Oxford England). 2010;26(12):1572–3. 10.1093/bioinformatics/btq170. 10.1093/bioinformatics/btq170 PMC2881355 20427518

[CR36] Xia Y, Rao L, Yao H, Wang Z, Ning P, Chen X. Engineering macrophages for Cancer Immunotherapy and Drug Delivery. Adv Mater (Deerfield Beach Fla). 2020;32(40):e2002054. 10.1002/adma.202002054. 10.1002/adma.202002054 32856350

[CR32] Xu S, Tang L, Li X, Fan F, Liu Z. Immunotherapy for glioma: current management and future application. Cancer Lett. 2020;476:1–12. 10.1016/j.canlet.2020.02.002. 32044356 10.1016/j.canlet.2020.02.002

[CR39] Yeo AT, Rawal S, Delcuze B, Christofides A, Atayde A, Strauss L, et al. Single-cell RNA sequencing reveals evolution of immune landscape during glioblastoma progression. Nat Immunol. 2022;23(6):971–84. 10.1038/s41590-022-01215-0. 35624211 10.1038/s41590-022-01215-0 PMC9174057

[CR25] Yoshihara K, Shahmoradgoli M, Martínez E, Vegesna R, Kim H, Torres-Garcia W, et al. Inferring tumour purity and stromal and immune cell admixture from expression data. Nat Commun. 2013;4:2612. 10.1038/ncomms3612. 24113773 10.1038/ncomms3612 PMC3826632

[CR29] Yu G, Wang LG, Han Y, He QY. clusterProfiler: an R package for comparing biological themes among gene clusters. OMICS. 2012;16(5):284–7. 10.1089/omi.2011.0118. 22455463 10.1089/omi.2011.0118 PMC3339379

[CR46] Zarember KA, Godowski PJ. Tissue expression of human toll-like receptors and differential regulation of toll-like receptor mRNAs in leukocytes in response to microbes, their products, and cytokines. J Immunol (Baltimore Md: 1950). 2002;168(2):554–61. 10.4049/jimmunol.168.2.554. 10.4049/jimmunol.168.2.554 11777946

[CR16] Zhao Z, Zhang KN, Wang Q, Li G, Zeng F, Zhang Y, et al. Chinese Glioma Genome Atlas (CGGA): a Comprehensive Resource with functional genomic data from Chinese glioma patients. Genom Proteom Bioinform. 2021;19(1):1–12. 10.1016/j.gpb.2020.10.005. 10.1016/j.gpb.2020.10.005 PMC8498921 33662628

