# Supplementary material for: Crosstalk between CD180-overexpression macrophages and glioma cells worsens patient survival through malignant phenotype promotion and immunosuppressive regulation
Source: Mol Med. 2024 Dec 20;30:264. doi: 10.1186/s10020-024-01029-w (PMC11660626; doi:10.1186/s10020-024-01029-w)
Supplement: Supplementary file 1 — Supplementary Material 1 [file 10020_2024_1029_MOESM1_ESM.docx]

**Supplementary materials**

**Supplementary Tables**

| Table S1. Primers for qRT-PCR. | |
| --- | --- |
| Gene | Sequence |
| CD180-F | AACCTAAGCCTGAACTTCAATGG |
| CD180-R | GCCAGAGAGACTGAGTAGTAGAG |
| GAPDH-F | ACGGATTTGGTCGTATTGGG |
| GAPDH-R | GGGATCTCGCTCCTGGAAG |

| Table S2. Antibodies used for western blot assay. | | |
| --- | --- | --- |
| Protein name | Brand | Lot Number |
| GAPDH | abcam | ab181602 |
| Snail | Abclonal | A5243 |
| Twist | Abclonal | A3237 |
| E-Cadherin | CST | 3195 |
| N-Cadherin | CST | 13116 |
| Flag | Abcam | ab205606 |

**Supplementary Figures**


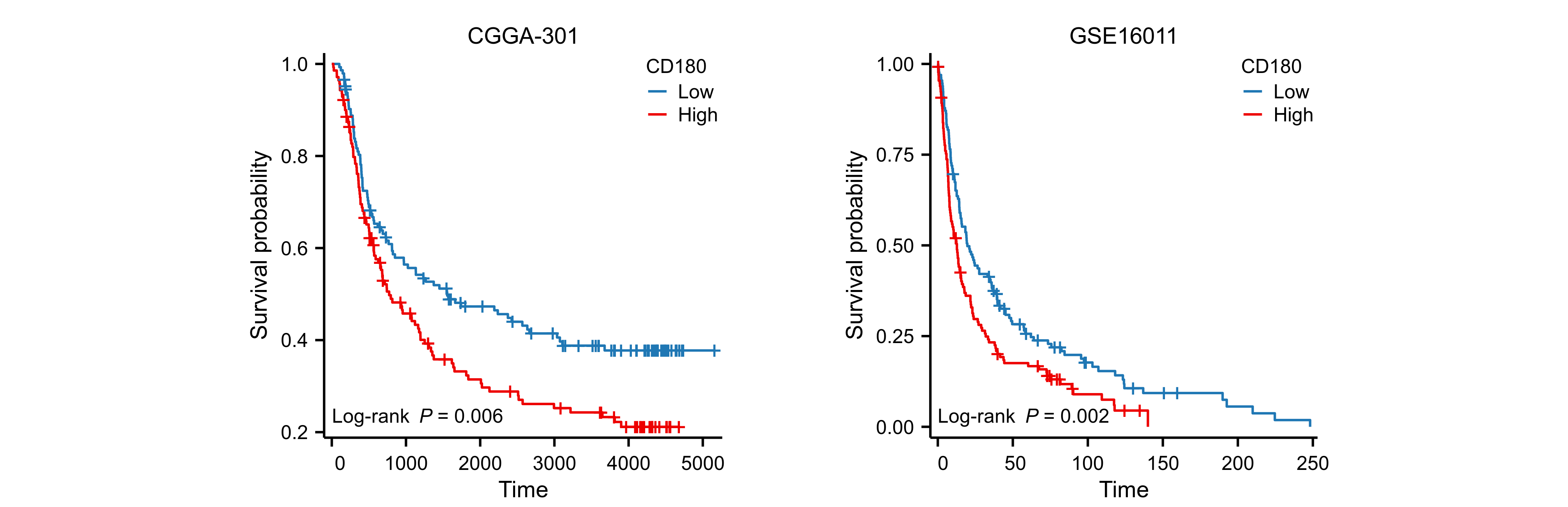


Fig. S1. Kaplan-Meier analysis in CGGA-301and GSE16011 datasets.


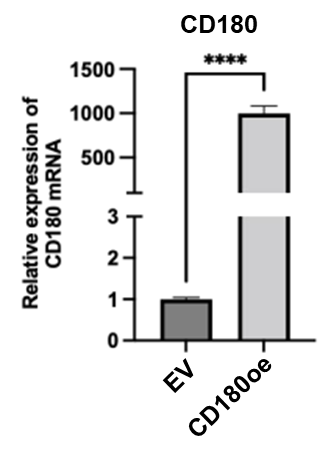


Fig. S2. qRT-PCR in macrophages transfected with CD180 overexpressing adenovirus compared with empty vehicles. ****, P<0.0001.


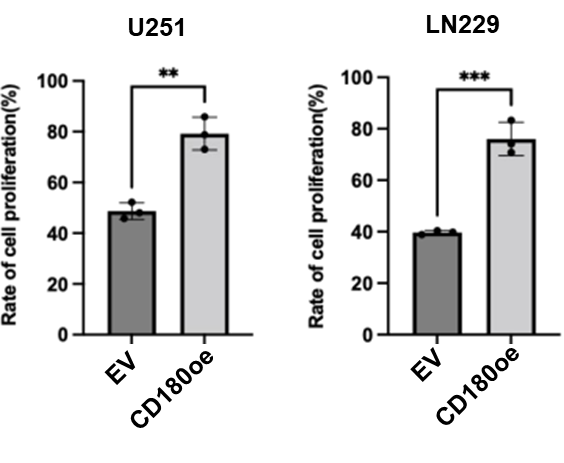


Fig. S3. Bar graph of Edu assay in U251 and LN229 cell lines. Scale bar, 50 μm. ***, P<0.001. Each experiment is replicated for three times.


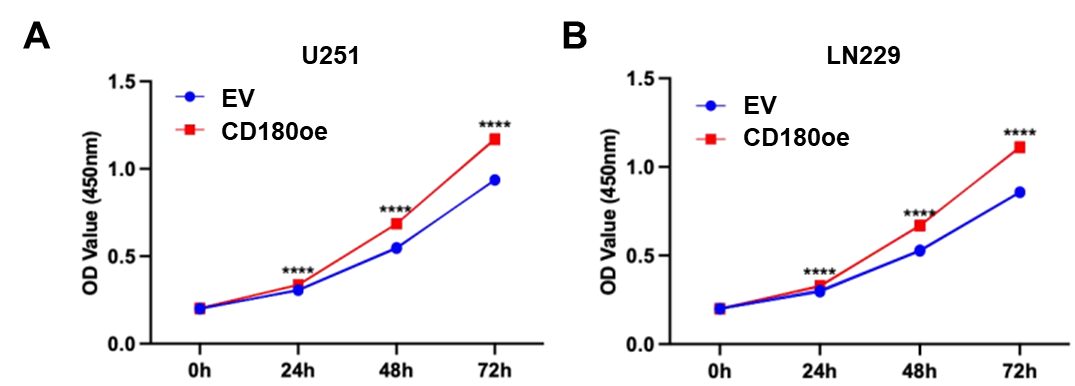


Fig. S4. Line graph shows the CCK-8 assays at 24 hours, 48 hours and 72 hours after co-culture in U251 (A) and LN229 (B) cell line.


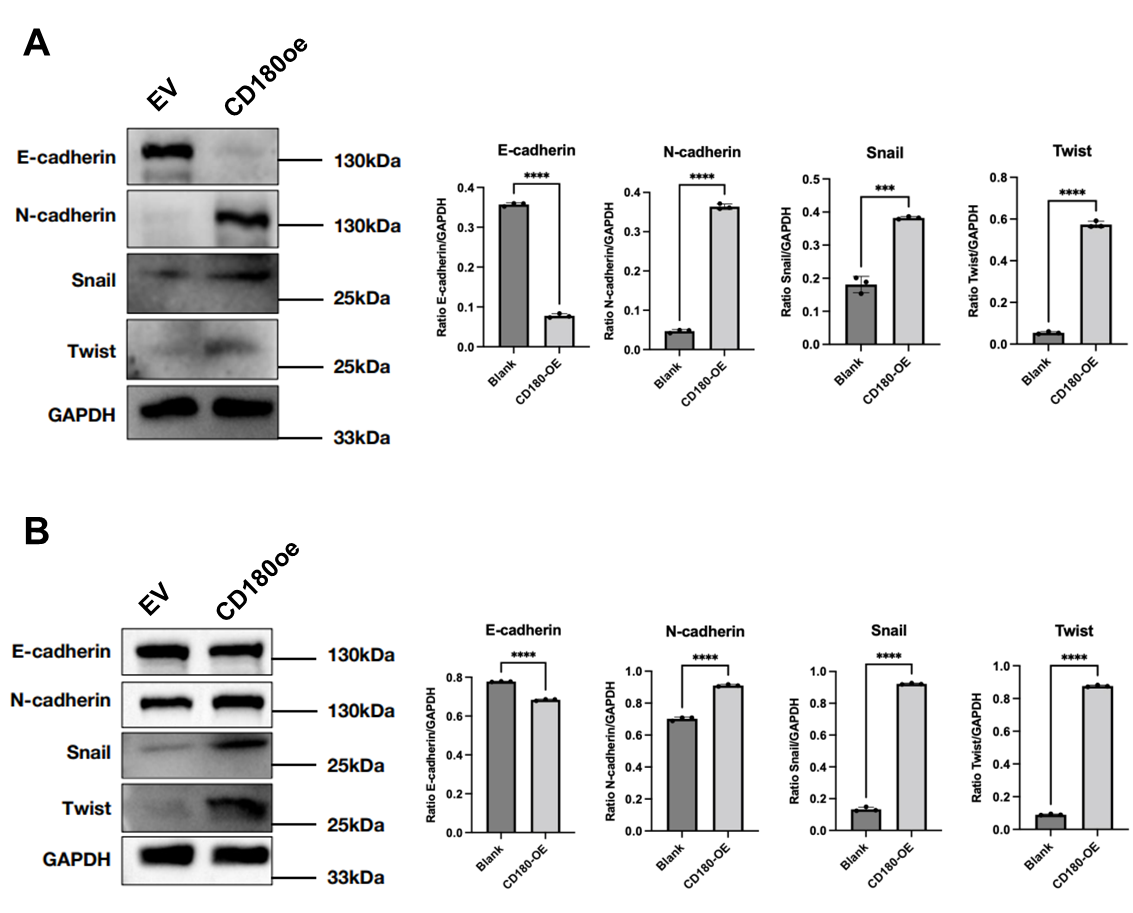


Fig. S5. Western blot images and the histograms of E-cadherin, N-cadherin, Snail, and Twist in U251 (A) and LN229 (B) cell line subjected to different treatments.


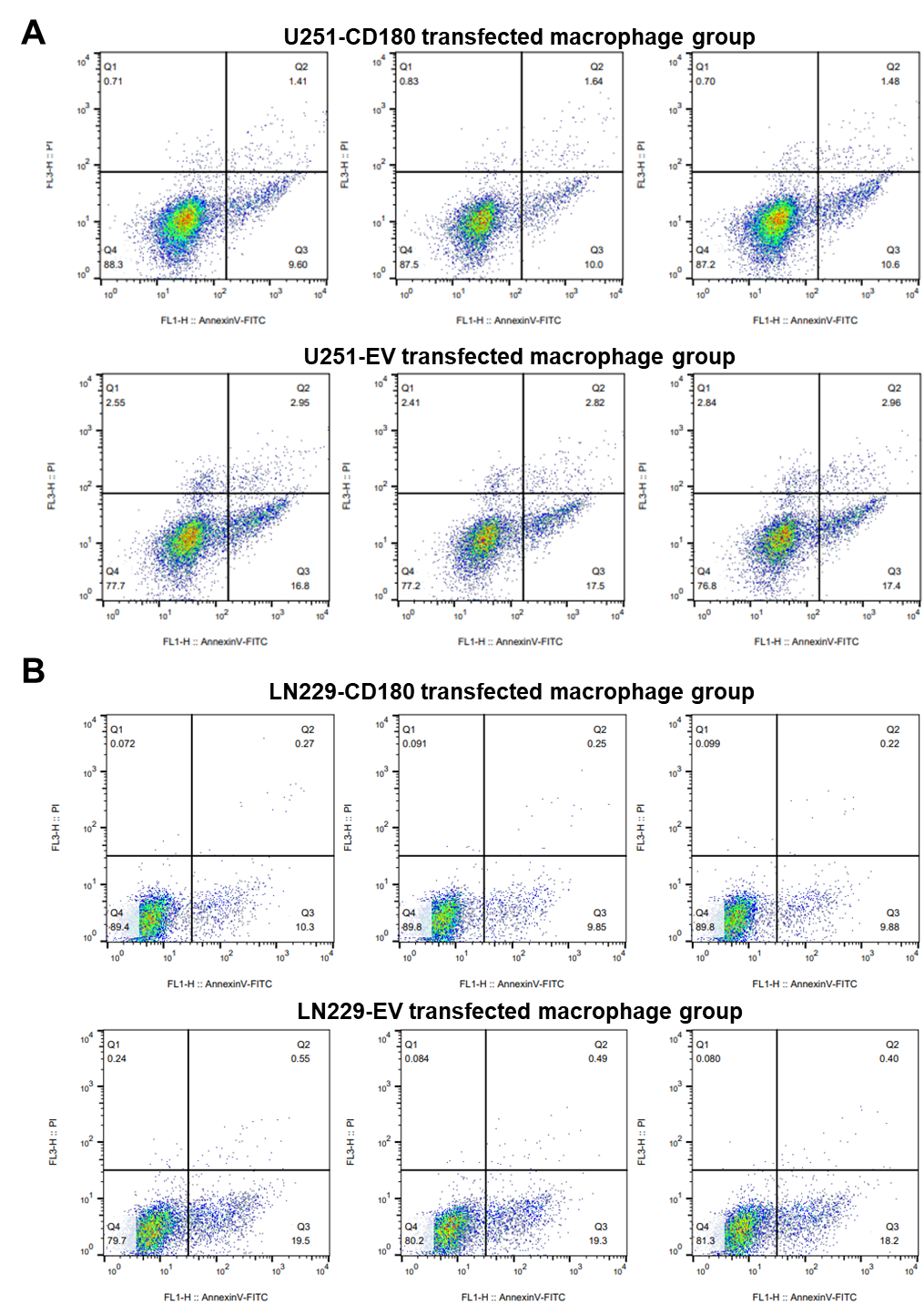


Fig. S6. Annexin V-PI flow cytometry analysis in U251 and LN229 cell lines before and after co-culture.


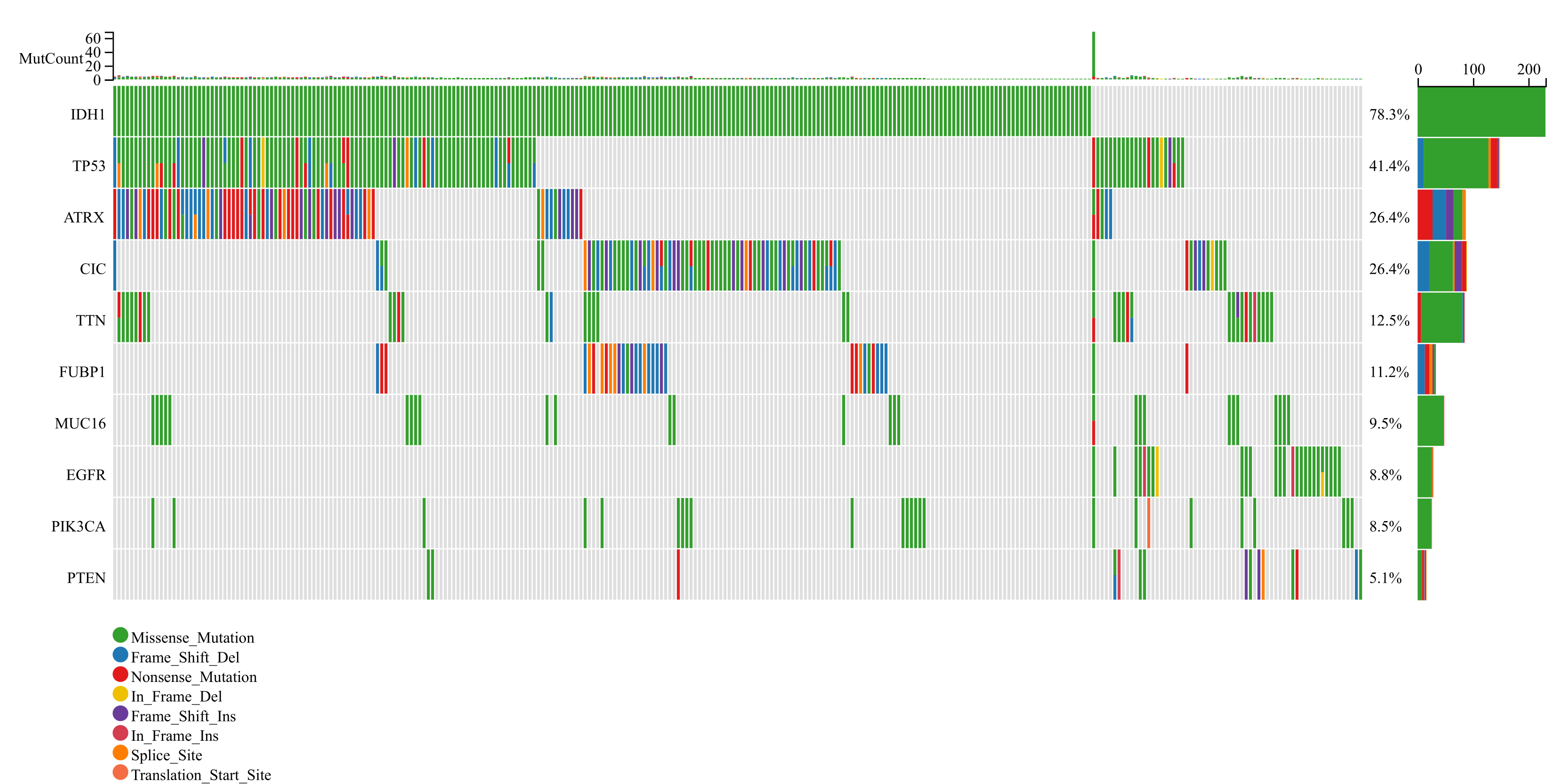


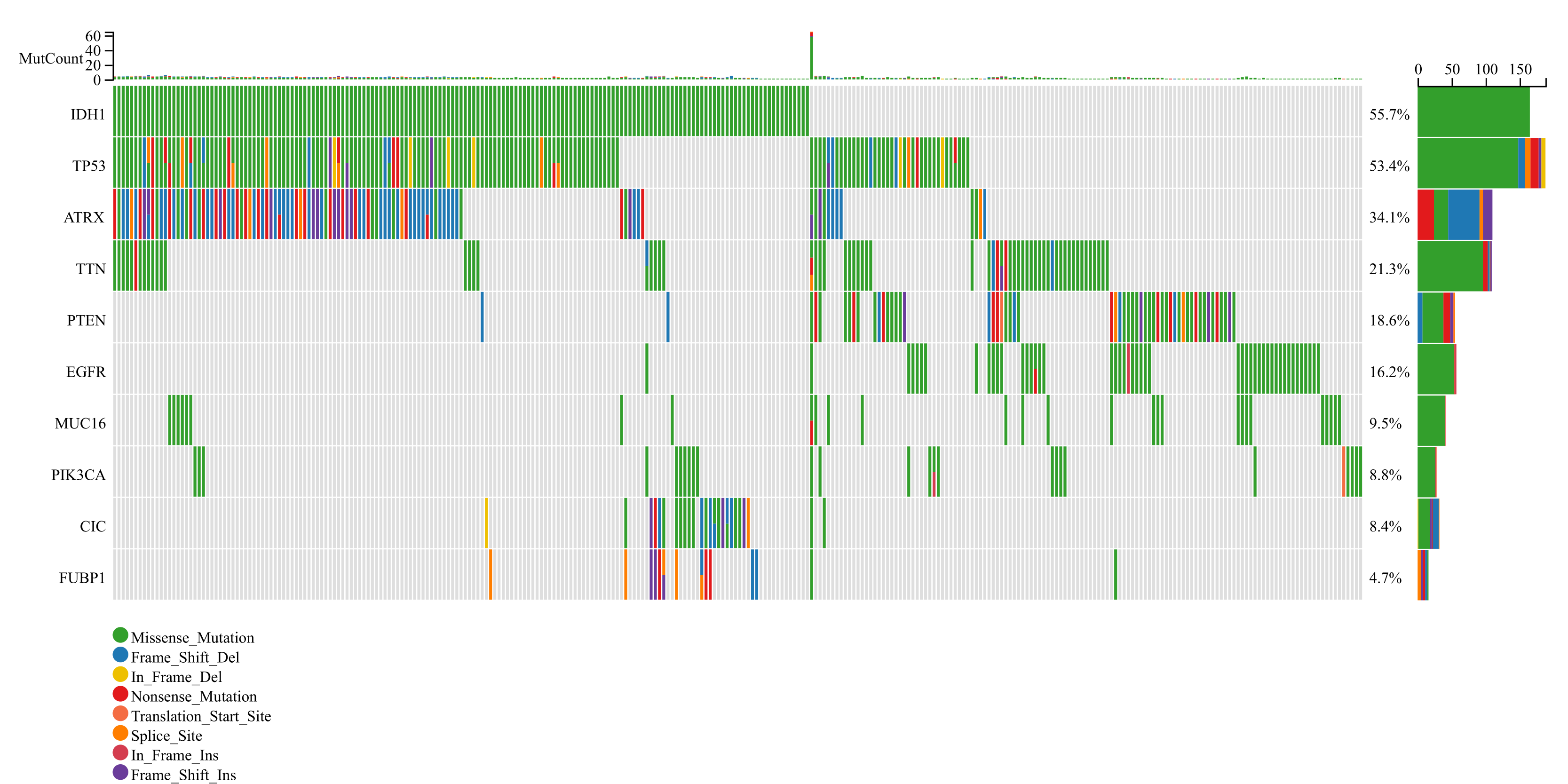


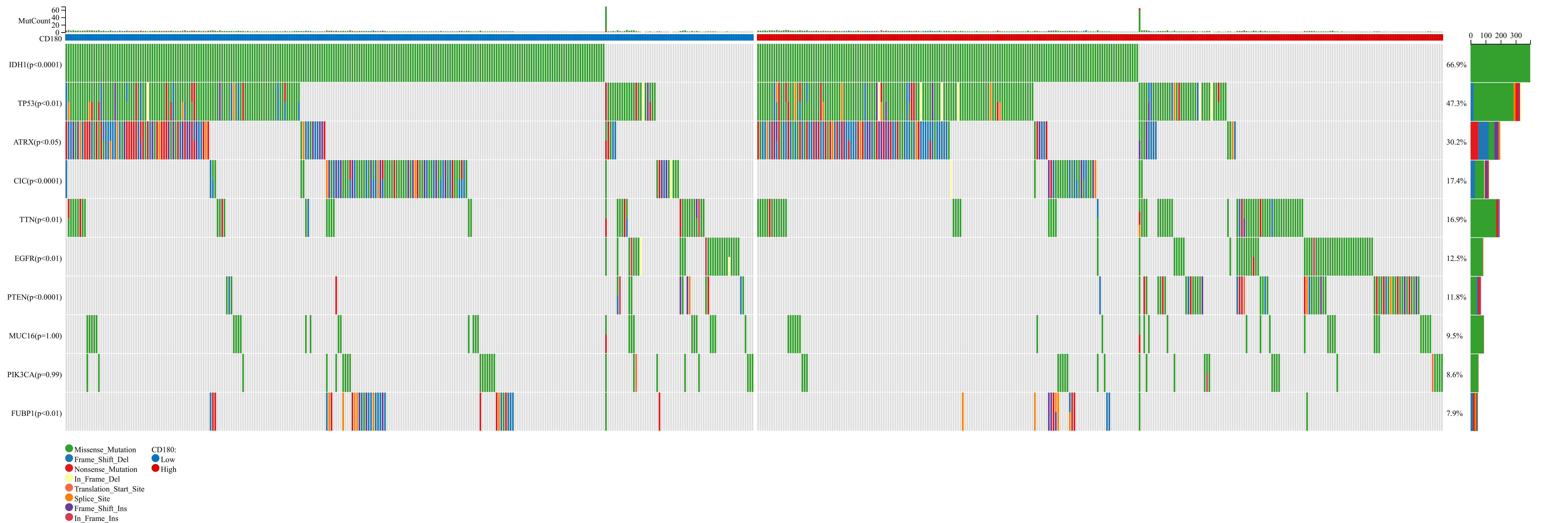


Fig. S7. Mutation characteristics of low and high CD180 expression groups and the prevalence comparison of the top 10 mutant genes in glioma.
